# Driving Electrochemical Organic Hydrogenations on Metal Catalysts by Tailoring Hydrogen Surface Coverages

**DOI:** 10.1021/jacs.4c15821

**Published:** 2025-04-08

**Authors:** Anna Ciotti, Motiar Rahaman, Celine Wing See Yeung, Tengfei Li, Erwin Reisner, Max García-Melchor

**Affiliations:** † School of Chemistry, CRANN and AMBER Research Centres, Trinity College Dublin, College Green, Dublin 2, Ireland; ‡ Yusuf Hamied Department of Chemistry, University of Cambridge, Lensfield Road, Cambridge CB2 1EW, United Kingdom; § School of Chemistry and Environment, Manchester Metropolitan University, Chester Street, Manchester M1 5GD, United Kingdom; ∥ Center for Cooperative Research on Alternative Energy (CIC energiGUNE), Basque Research and Technology Alliance (BRTA), Alava Technology Park, Albert Einstein 48, 01510 Vitoria-Gasteiz, Spain; ⊥ IKERBASQUE, Basque Foundation for Science, Plaza de Euskadi 5, 48009 Bilbao, Spain

## Abstract

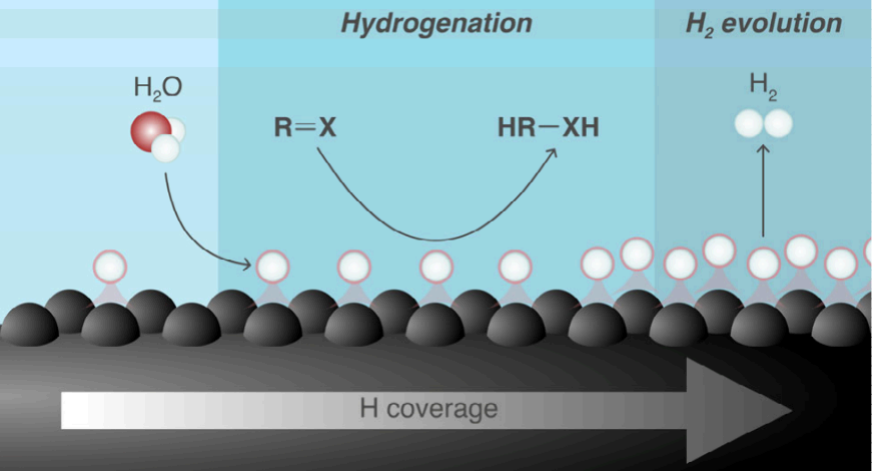

Electrochemical hydrogenation, powered by renewable electricity, represents a promising sustainable approach for organic synthesis and the valorization of biomass-derived chemicals. Traditional strategies often rely on alkaline conditions to mitigate the competing hydrogen evolution reaction, posing challenges in sourcing hydrogen atoms for hydrogenation, which can be addressed through localized water dissociation on the electrode surface. In this study, we present a computationally guided design of electrochemical hydrogenation catalysts by optimizing hydrogen coverage density and binding strength on the electrode. Our theoretical investigations identify Cu, Au, and Ag − metals with moderate hydrogen coverage − as promising catalysts for electrochemical hydrogenations in alkaline media. These predictions are experimentally validated using a model organic substrate (acetophenone), achieving yields and faradaic efficiencies of up to 90%. Additionally, Cu, a nonprecious metal, is demonstrated to selectively hydrogenate a wide range of unsaturated compounds, including C=O, C=C, C≡C, and C≡N bonds, at low potentials with moderate to excellent conversion rates and chemoselectivities. This work highlights the potential of tailoring hydrogen coverage on electrode surfaces to rationally design nonprecious metal electrocatalysts for efficient organic hydrogenations. The insights gained here are expected to inform the development of more effective catalysts for organic hydrogenations and other industrially relevant chemical transformations.

## Introduction

Since the industrial revolution, anthropogenic emissions of CO_2_, partly driven by industrial chemical processes that depend on fossil fuel-derived reagents, have had a lasting impact on the Earth’s environment and climate.^1^ Transitioning to a defossilized economy can be facilitated by electrifying industrial synthetic processes, particularly through the integration of electrosynthesis with renewable energy sources.^2−6^ One promising approach in this regard is the electrochemical hydrogenation (ECH) of organic substrates, which offers a sustainable pathway for synthesizing organic compounds and valorizing biomass derivatives into valuable fine chemicals, natural products, and pharmaceuticals.^7−11^ Moreover, if powered by renewable electricity under mild conditions, ECH has the potential to replace traditional thermal hydrogenation processes, which typically require purification of H_2_ from steam methane reforming, as well as elevated pressures and temperatures.^12−14^

**Figure 1:**
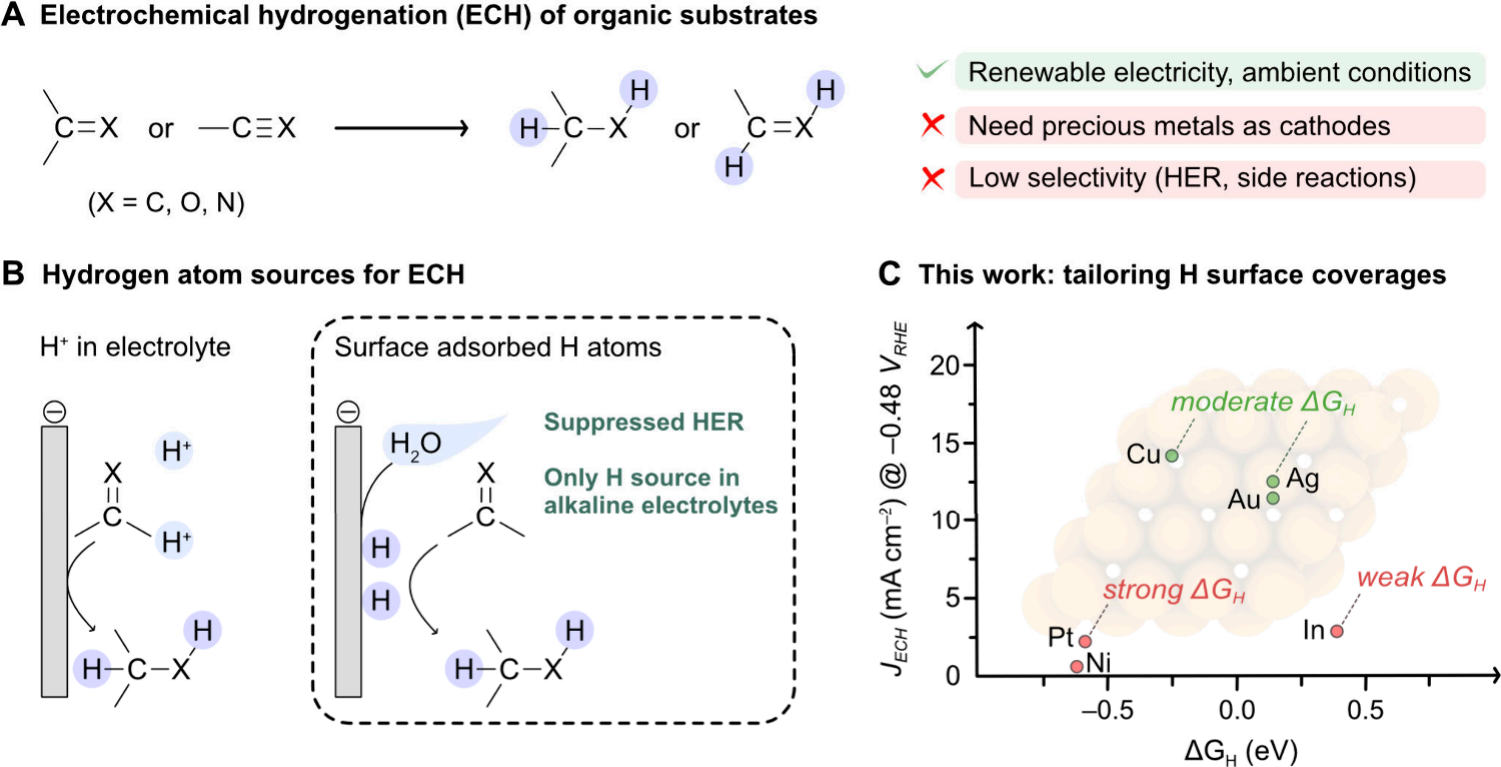
Electrochemical hydrogenation (ECH) of organic substrates. (A) Summary of state-of-the-art in ECH of organic compounds, emphasizing the main advantages and challenges. (B) Schematic illustration of the hydrogen source for ECH in acidic versus alkaline conditions. (C) Schematic representation of ECH driven by the tailoring of hydrogen surface coverage, as demonstrated in this study. The *y*-axis represents the partial current density toward ECH (*J*_*ECH*_), and the *x*-axis shows the Gibbs energy of hydrogen adsorption (*ΔG*_*H*_). In the background: Illustration of a metal cathode surface with 75% of the face-centered cubic (*fcc*) sites covered by hydrogen atoms.

ECH reactions have been demonstrated across a variety of functional groups, including C=O, C=C, C≡C, and C≡N (Figure 1A).^15−28^ However, these systems often rely on precious metal catalysts, such as Pt and Pd.^22,29,30^ Recently, there has been a growing interest in using nonprecious metal catalysts like Cu, Ni, and Ag for organic ECH.^16−18,24,25,31−44^ Despite these advances, a significant challenge in aqueous media is the competition with the more facile hydrogen evolution reaction (HER), which typically requires less negative potentials and thus compromises the faradaic efficiency (FE) for the desired ECH products. A viable strategy to mitigate this competition is to operate in alkaline media, where the proton concentration is significantly lower.^45^ For instance, in the ECH of acetonitrile, a FE of 80–90% was achieved at 100–500 mA cm^–2^ in 1 M NaOH, a stark contrast to the dominant HER observed under acidic or neutral conditions.^18^

Alkaline conditions favor ECH by suppressing HER, but they also introduce new challenges. In acidic media, H atoms are directly sourced from hydronium ions in the electrolyte. In contrast, in alkaline solution, H atoms are primarily derived from water dissociation on the electrode surface (Figure 1B), provided the corresponding kinetic barrier is lower than that for direct hydrogenation of the reactant.^46^ According to the Sabatier’s principle,^47^ these adsorbed H atoms must exhibit moderate binding strength at the applied potential to effectively facilitate ECH of organic substrates. Therefore, we hypothesized that maintaining a relatively high density of weakly bound H atoms is crucial for promoting efficient ECH in alkaline media while minimizing HER.

Recent computational studies have provided insights into the ECH of organic compounds on Pd systems, primarily by analyzing the binding energies of key reaction intermediates.^24,25^ While previous literature reports have demonstrated the active role of surface coverages in a variety of electrocatalytic reactions such as CO_2_ reduction, NO reduction, HER, and ECH,^48−52^ to the best of our knowledge, this is the first example of the rational design of ECH electrocatalysts achieving excellent catalytic activity and selectivity across a broad range of substrates through tailored H coverages. We believe that understanding this aspect is critical for the strategic development of high-performance ECH electrocatalysts based on earth-abundant elements, and the lack of such knowledge may explain why these materials have remained elusive.

Herein, we present a bottom-up approach for the design of nonprecious metal cathodes optimized for the selective ECH of organic substrates. Through computational investigations, we analyzed the density and binding strength of hydrogen surface coverages on various transition metals under relevant ECH conditions, identifying Cu, Au, and Ag as particularly promising electrocatalysts. These theoretical predictions were validated experimentally in the ECH of acetophenone (AP) to 1-phenylethanol (1-PEA), achieving FEs and yields up to 90% at low applied potentials (ca. −0.5 V vs the reversible hydrogen electrode, RHE) in alkaline media (pH ∼ 12). Remarkably, these cathodes demonstrated superior performance compared to other transition metals by at least an order of magnitude in terms of FEs and yields, including In, Ni, and even the precious Pt metal, which is considered a state-of-the-art ECH electrocatalyst (Figure 1C).^20,21^ Furthermore, our experimental results show that this bottom-up approach can be generalized to a wide range of unsaturated organic substrates with C=O, C=C, C≡C, and C≡N bonds, achieving impressive yields (70–90%) on Cu electrodes. Overall, this work highlights the critical role of hydrogen surface coverages in ECH processes and demonstrates how this insight can be harnessed to design non-noble metal cathodes for the sustainable production of chemical feedstocks and value-added products.

## Results and Discussion

### Metal Surface Coverages under ECH Conditions

While alkaline electrolysis can minimize the competing HER, the electrode surface under these conditions might still facilitate water hydrolysis to provide the H atoms necessary for ECH. We hypothesized that an ideal ECH catalyst should not only promote water dissociation in an alkaline medium but also retain H atoms in a configuration that minimizes HER while maintaining moderate binding strength to effectively hydrogenate organic substrates. To identify cathode materials meeting these criteria, we conducted periodic density functional theory (DFT) calculations on several transition metals, specifically Ag, Au, Cu, Ni, and In (details available in the Supporting Information). These elements were chosen due to their varying H binding energies at reducing potentials, as reported in previous theoretical studies on HER.^53^ For comparison, we included Pt in our investigation, given its status as a state-of-the-art ECH electrocatalyst.^20,21^

**Figure 2:**
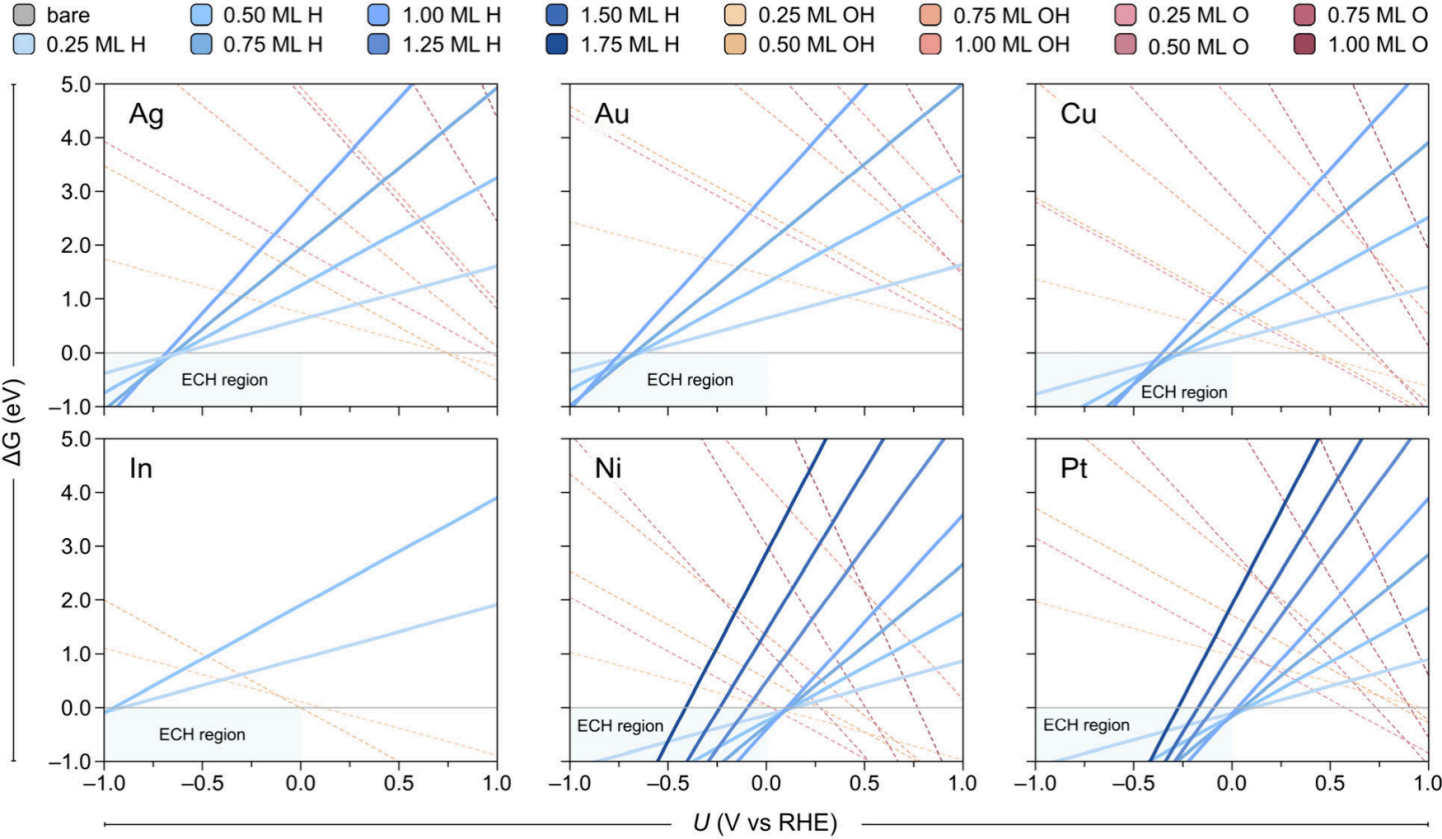
Surface coverage analysis of Ag, Au, Cu, Ni, In, and Pt metal cathodes. The plots show relative Gibbs energies of surface terminations with varying concentrations of adsorbed *H, *OH, and *O species (* denotes a metal surface site) as a function of applied potential vs RHE, *U*. The dashed red and orange lines represent *OH and *O coverages, respectively, while the thick blue lines denote *H coverages, and the gray line indicates the bare surface. Darker shades correspond to higher coverage densities. The region relevant to ECH is highlighted in light blue. All metals were modeled as (111) surface slabs with *p*(2 × 2) periodicity, except for In, which was modeled as a *p*(1 × 2)-(101) slab. Different colors and labels indicate the relative concentration of adsorbed species in each surface termination, with darker shades representing higher densities. As each supercell contains four surface sites (*bridge*, *fcc*, *hcp* and *top* for *fcc* metals, and *top*, *bridge*, and *hollow*, with or without a subsurface metal atom, for In), the fraction of occupied sites varies in multiples of 0.25. For instance, on *fcc* metals, 0.25 monolayer (ML) H denotes a quarter of the *fcc* sites covered by H atoms, 0.50 ML H indicates half of the *fcc* sites covered, etc. Further details can be found in the Supporting Information.

For each metal, we examined the relative Gibbs adsorption energies (*ΔG*_*i*_) of different surface terminations with varying concentrations of *H, *OH, and *O species (* denotes a surface metal site) as a function of applied potential. The results of this analysis are illustrated in the surface diagrams depicted in Figure 2, which show that *OH and *O coverages (orange/red lines) are favored at positive (oxidizing) potentials, while *H coverages (blue lines) are favored at negative (reducing) potentials, as expected. Within the potential window typically used in ECH studies (i.e., from 0 to –1.0 V vs RHE), the metals display different concentrations of adsorbed H atoms. The calculated *ΔG*_*H*_ (in eV) for a single H atom adsorbed on the different metals at 0 V follows the trend: In (+0.90) > Ag (+0.64) ∼ Au (+0.64) > Cu (+0.25) > Pt (−0.09) > Ni (−0.12). These values not only describe HER activity,^54^ but also indicate the thermodynamic driving force for water dissociation and ECH.^46,54^ Specifically, very positive *ΔG*_*H*_ values suggest a lack of surface H atoms for ECH (e.g., In, which displays the highest *ΔG*_*H*_ value, is predicted to present a mostly bare surface), whereas negative values, while favoring water dissociation, could hinder ECH by making the H transfer to the substrate energetically unfavorable.^46^ Based on these computational data, and considering that *ΔG*_*H*_ shifts by +*eU* with the applied potential *U* according to the computational hydrogen electrode model,^55^ we identified Cu, Ag, and Au as promising candidates for promoting ECH of organic substrates in alkaline media at relatively low potentials, by sourcing H atoms from the surface coverage.

### ECH of Acetophenone on Different Metal Cathodes

**Figure 3:**
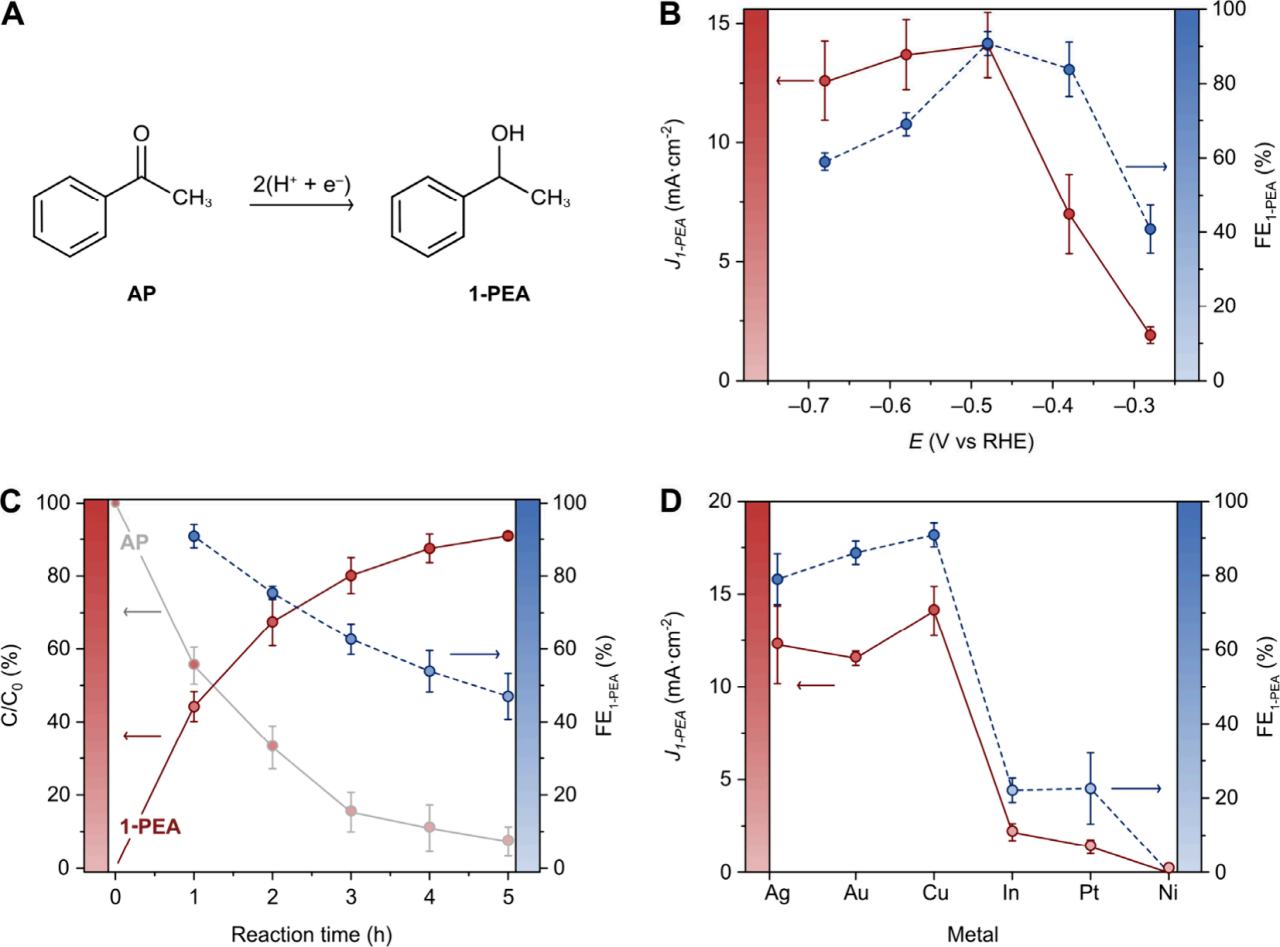
ECH of AP to 1-PEA. (A) Schematic representation of the hydrogenation of AP to 1-PEA. (B) *J*_1-PEA_ (red, solid line) and FE_1-PEA_ (blue, dashed line) for the ECH of AP to 1-PEA measured on the Cu electrocatalyst at potentials between −0.28 and −0.68 V vs RHE after 1 h electrolysis. (C) Concentration profile of products (red, solid line) and reactants (gray, solid line), and FE_1-PEA_ (blue, dashed line) for the ECH of AP on the Cu electrocatalyst over 5 h of electrolysis at −0.48 V vs RHE. *C* and *C*_*0*_ indicate the AP (gray trace) and 1-PEA (red trace) concentration at the time of interest and the initial AP concentration, respectively. Concentrations of reactants and products were quantified by ^1^H NMR. (D) *J*_1-PEA_ (red trace) and FE_1-PEA_ (blue trace) were measured for the ECH of AP to 1-PEA on Ag, Au, Cu, In, Pt, and Ni electrocatalysts at −0.48 V vs RHE after 1 h electrolysis. Error bars correspond to the standard deviation of triplicate (*n* = 3) experiments. Lines are shown as guides to the eye.

To validate the trends predicted by our theoretical studies, we performed ECH experiments using AP as a model substrate, chosen because: (i) the C=O group is activated by the aromatic structure, and (ii) the hydrogenation of AP is a common route for producing 1-PEA (Figure 3A), an important precursor in pharmaceutical and fragrance industries.^56^ Among the metal cathodes investigated, Cu was first examined, as DFT calculations predicted it to be one of the most active at relatively low potentials. The Cu electrocatalyst was prepared by a H_2_ bubble template-assisted galvanostatic electrodeposition method following a previously reported protocol (see Supporting Information).^57^ Scanning electron microscopy (SEM) analysis revealed a porous dendritic morphology (Figure S1), and powder X-ray diffraction (XRD) analysis confirmed the crystal planes of the Cu catalyst (Figure S2).

Electrochemistry experiments were performed in an aqueous potassium phosphate buffer solution (pH = 11.8). Details of the experimental setup can be found in the Supporting Information. Cyclic voltammograms (CVs) collected on the Cu electrocatalyst (Figures S3 and S4) showed a significant increase in current density upon the addition of AP to the electrolyte solution, indicating Cu catalysts’ capability to promote ECH. Chronoamperometry experiments were conducted within a potential window of −0.28 V and −0.68 V vs RHE, and the reaction mixture was analyzed by proton nuclear magnetic resonance (^1^H-NMR) spectroscopy after 1 h to quantify the yield of 1-PEA. The FE and partial current density toward 1-PEA (FE_1-PEA_ and *J*_1-PEA_, respectively) are presented in Figure 3B. At −0.28 V vs RHE, a FE_1-PEA_ of 41 ± 6% and *J*_1-PEA_ of 1.9 ± 0.4 mA cm^–2^ were observed. These values increased with more negative potentials, reaching maximum values of 91 ± 3% and 14 ± 1 mA cm^–2^ at −0.48 V vs RHE. The remaining product was confirmed to be H_2_ by gas chromatography (GC), which led to the decrease of FE_1-PEA_ and *J*_1-PEA_ at higher potentials, between −0.58 and −0.68 V vs RHE. The total current density (*J*_total_) and partial current density for H_2_ (*J*_H2_) are shown in Figure S6, along with the calculated *ΔG*_*H*_ (per H atom) for the lowest energy surface coverages at various potentials. These results, together with those presented in Figure 3B, confirm that the competing HER becomes more favorable at potentials below −0.5 V vs RHE, accounting for the observed changes in ECH activity. Optimization of the electrolyte pH and Cu catalyst deposition time (which influences the catalyst’s thickness and surface pore diameter) are shown in Figure S7, revealing an optimal pH of 11.8 and a deposition time of 40 s based on the measured FE_1-PEA_ and *J*_*1-PEA*_ values.

Figure 3C presents the results from controlled potential electrolysis of AP at −0.48 V vs RHE over 5 h under stirring (10 mL of solution containing 0.5 mmol AP, geometrical electrode surface area = 0.84 cm^2^). The reaction mixture was analyzed by ^1^H-NMR spectroscopy (Figure S8) every hour to quantify the amounts of AP and 1-PEA in the solution. After 5 h, a yield of 91 ± 1% was achieved for 1-PEA, with only 7.3 ± 4% of AP remaining in the electrolyte solution. The FE_1-PEA_ was above 60% during the first 3 h of electrolysis and was maintained at 47 ± 6% when the conversion was completed after 5 h. The ECH of AP was also investigated on Ag, Au, In, Pt, and Ni electrocatalysts (Figure S9 shows their morphologies) at −0.48 V vs RHE for 1 h (Figure 3D). Ag and Au exhibited high FE_1-PEA_ (80–90%) and *J*_*1-PEA*_ (10–13 mA cm^–2^), whereas In and Pt showed much lower performances (i.e., FE_1-PEA_ ∼ 20% and *J*_*1-PEA*_ ≤ 2.5 mA cm^–2^), and Ni did not show any ECH activity. Optimization of the potential on Ag, Au, and Pt (Figure S10) revealed a similar trend to that observed for Cu, except that Ag and Au displayed an optimal potential shifted cathodically to −0.68 V vs RHE. These results are consistent with our DFT investigations in Figure 2, which predict that the hydrogen coverage on Ag and Au increases at more negative potentials (in the range of −0.5 and −0.75 V vs RHE) while exhibiting moderate *ΔG*_*H*_ values − two conditions necessary for efficient ECH. These findings collectively demonstrate the efficient ECH of AP to 1-PEA on Cu, Ag, and Au, as predicted by computational surface coverage studies.

### ECH Mechanistic Studies

#### Resting States of Metal Cathodes

To further elucidate the reactivity trends observed in our experiments, we investigated the reaction mechanism for the ECH of AP to 1-PEA on various metal cathodes using DFT calculations. Given that the p*K*_a_ of the monohydrogenated form of AP (APH·) is reported to be of 9.9 in water,^58^ we assumed that the reduced form of AP (AP^–^·) could not efficiently accept protons from the solvent at the working pH of 11.8. Consequently, we posited that the H atoms required for ECH must be primarily sourced from the H surface coverage, if present.

**Figure 4:**
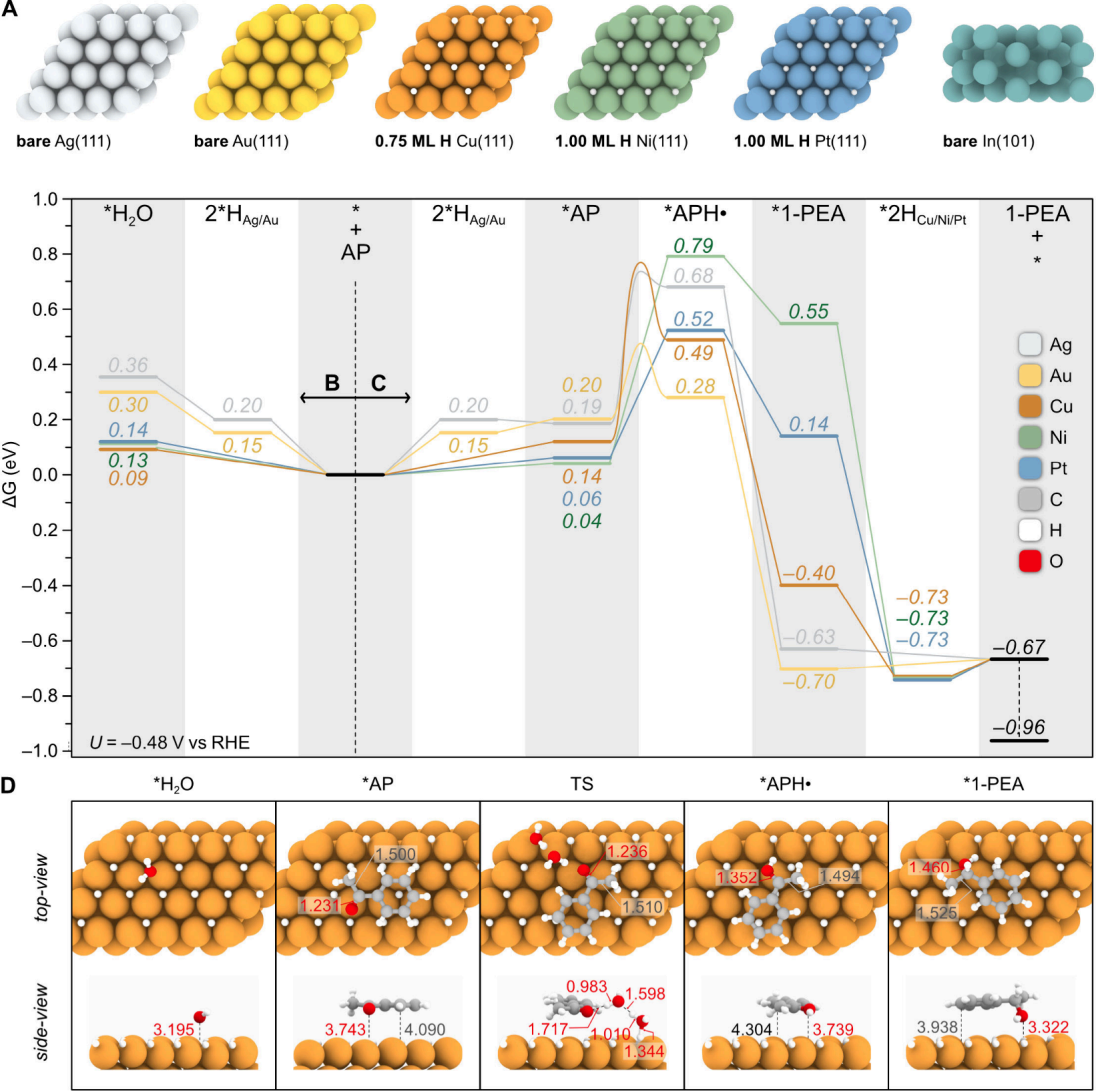
ECH mechanistic studies on different metal cathodes. (A) Resting states of Ag, Au, Cu, Ni, Pt, and In metal cathodes under ECH conditions based on the surface coverage analysis shown in Figure 2. (B) Gibbs energy for water adsorption on the metal electrodes, computed relative to a water bilayer (see Supporting Information for details). (C) Gibbs energy profile for the ECH of AP to 1-PEA calculated at the experimental potential of −0.48 V vs RHE, computed according to eqs S9–S19. Notably, the hydrogenations of AP to APH· and 1-PEA via the transfer of surface-bound *H species are purely chemical processes. As such, their energies and the reported kinetic barriers, referenced to the adsorbed *AP, remain independent of the applied potential. In contrast, the electrochemical generation of surface *H is potential-dependent and is promoted under increasingly negative bias. On Ag(111) and Au(111), the ECH mechanism involves the reduction of two protons (2*H_Ag/Au_), AP adsorption (*AP), hydrogenation at the O atom (*APH·), hydrogenation at the C atom (*1-PEA), and product desorption (1-PEA). On Cu(111), Ni(111), and Pt(111), the ECH mechanism involves AP adsorption (*AP), hydrogenation at the O (*APH·), hydrogenation at the C (*1-PEA), refilling of the H vacancies (2*H_Cu/Ni/Pt_), and product desorption (1-PEA). The energy of 1-PEA corrected by its estimated gas-phase error with respect to AP is also shown with a dotted line (see Supporting Information for details). (D) Top and side view representations of the optimized structures of physisorbed *H_2_O and the main reaction intermediates *AP, *APH·, and *1-PEA on Cu(111). The TS for the first *AP hydrogenation at the O atom, assisted by two water molecules on the diffused 0.75 ML H coverage of Cu, is also shown. Relevant bond distances (in Å) involving C and O atoms are shown in gray and red, respectively.

As discussed earlier, DFT calculations predict the In(101) surface to be mostly bare under reaction conditions due to its endergonic *ΔG*_*H*_ (+0.42 eV at −0.48 V vs RHE). This prediction accounts for the relatively poor ECH activity observed on In, likely due to the lack of sufficient surface H atoms, and thus no further mechanistic investigations were conducted for this cathode. In contrast, despite their slight endergonic *ΔG*_*H*_ values, we assessed the ECH activity of *p*(6 × 4) Ag(111) and Au(111) supercells, as their *ΔG*_*H*_ is much closer to thermoneutral under reaction conditions (ca. +0.2 eV on a *p*(2 × 2) supercell with a 0.25 ML H coverage at −0.48 V vs RHE), suggesting that water hydrolysis could be feasible at room temperature. However, these pristine metals require the binding of two hydrogen atoms necessary for the ECH of AP (2*H_Ag/Au_ in Figure 4B–C, see Supporting Information for details), resulting in a moderately endergonic step. Notably, the higher *J*_1-PEA_ and FE_1-PEA_ values observed for Ag and Au at more negative potentials (Figure S10) align with enhanced hydrogen coverage predicted by DFT calculations (Figure 2), which is crucial to sustain the ECH process. For Cu, Ni, and Pt, the ECH mechanism was explored on the surface coverages predicted at −0.48 V vs RHE in Figure 2, corresponding to 75% of the *fcc* sites covered by H on Cu (0.75 ML H) and 100% H coverage on Ni and Pt (1.00 ML H). The resting states for all the investigated metals are illustrated in Figure 4A.

#### Competition for Binding between AP and Water

Given that the presence of AP in solution has been shown to decrease the hydrogen coverage on a Pt electrode,^20^ we first investigated the competition between AP and water for binding on the electrode surface, hypothesizing that the hydrogen coverage depends not only on *ΔG*_*H*_ but also on the electrode’s accessibility to the aqueous electrolyte. Notably, the ability of catalyst surfaces to adsorb and dissociate water has been demonstrated to play a crucial role in promoting both HER and ECH processes.^45,46,59,60^ Analysis of AP binding via both the π-system and the carbonyl oxygen revealed that flat physisorption at approximately 3.5–4 Å through the π-system is the preferred binding mode on all metal cathodes. In comparison, a water molecule was found to adsorb more closely, at around 3 Å. Due to the weakly physisorbed nature of *AP (Figure 4D), the calculated binding energies relative to the hydrogen-covered surfaces are narrowly distributed, ranging from −0.01 eV (Ag) to +0.14 eV (Cu) (Figure 4C). Consistent with the literature,^20^ *AP binding is favored over water adsorption on the Pt(111) surface, a trend also observed on Ag, Au, and Ni metals (Figure 4B–C). Moreover, the larger size of AP likely allows it to displace more than one water molecule upon adsorption, increasing the energy gain and reducing the competing HER, as discussed in detail below.

#### ECH Reaction Mechanism

We next modeled the hydrogenation of *AP, which can occur either at the C or O atoms of the carbonyl group. According to our DFT calculations, hydrogenation at the O atom is thermodynamically preferred on all metals except Ag and Cu, which favor hydrogenation at the C atom (see Supporting Information). The transition state (TS) for the latter was therefore assessed in the presence of up to three explicit water molecules. Notably, we observed that the calculated energy barriers (*ΔG*^*‡*^) significantly decrease with the introduction of two water molecules, which act as a proton shuttle between the metal surface and the physisorbed *AP (TS in Figure 4D), while no marked improvement is observed with the addition of a third water molecule (Figure S11). Specifically, the energy barriers decreased by ca. 0.6 eV in the presence of two water molecules compared to the calculation featuring only one, highlighting the critical role of the water solvent in promoting ECH. Hydrogenation at the C atom, however, is hindered by a significant energy barrier (*≥*+2.0 eV, Figure S11), making this pathway unlikely under ambient experimental conditions.

Hydrogenation of the carbonyl O in *AP leads to the intermediate *APH·, a species with a radical character delocalized over the aromatic ring, as confirmed by the magnetic moments obtained in the DFT simulations (see Supporting Information for details). This delocalization stabilizes the intermediate and lowers its energy. However, this second step remains endergonic on all the investigated metals relative to the adsorbed *AP, with the following order: Au (+0.08 eV) < Cu (+0.35 eV) < Pt (+0.46 eV) < Ag (+0.49 eV) < Ni (+0.75 eV) (Figure 4C). Once *APH· is formed, hydrogenation at the C atom yields the final product, *1-PEA, in an exergonic process across all cathodes. The formation of *1-PEA relative to the catalyst resting state (*), which represents the thermodynamic driving force for the overall ECH reaction, follows this trend: Au (−0.70 eV) ∼ Ag (−0.63 eV) < Cu (−0.40 eV) < Pt (+0.14 eV) < Ni (+0.55 eV) (Figure 4C). This mirrors the order of the *ΔG*_*H*_ values for a single H atom adsorbed on the different metals at 0 V vs RHE (Ag ∼ Au > Cu > Pt > Ni), reinforcing the idea that hydrogen density and binding strength could serve as key reaction descriptors for ECH. Importantly, the overall energy profile for AP reduction on Ag and Au, which accounts for the energy cost of generating two reactive *H atoms, is consistent with ECH activity at room temperature. This finding further supports the mechanism of AP hydrogenation from surface *H on these two metals. Finally, on Cu(111), Ni(111), and Pt(111), the hydrogen coverage is regenerated (2*H_Cu/Ni/Pt_), followed by the desorption of 1-PEA, completing the catalytic cycle.

Overall, DFT calculations indicate that the ECH of AP to 1-PEA is thermodynamically favorable across all the investigated metals under the experimental conditions used in this study, with the first hydrogenation step to yield *APH· being the most endergonic in the reaction mechanism. Notably, Ni exhibits the most endergonic hydrogenation toward *APH·, which, coupled with the strong *ΔG*_*H*_ leading to a more facile HER,^54,61^ accounts for the very poor ECH performance observed experimentally. In the case of Pt, while the energy landscape for ECH is more favorable, its poor activity is attributed to the strong competition with HER, as discussed in detail below.^20,21^ In contrast, the energetics on Au, Ag, and Cu are compatible with catalytic activity at room temperature (Figure 4C), which aligns with experimental observations. Additionally, we find that H diffusion, which is reported to be facile on *fcc* metal surfaces,^62^ further lowers the ΔG_*APH·_ on the 0.75 ML H coverage on Cu from +0.49 eV to +0.39 eV (see Supporting Information). Moreover, the mechanistic investigation of AP hydrogenation on the 0.50 ML H and 1.00 ML H coverages on Cu, which exhibit similar stability to the 0.75 ML H, suggests comparable ECH activities based on their associated ΔG_*APH·_ values (see Figure S11).

#### Rationalizing ECH Activity

To further elucidate the ECH mechanism on the Au, Ag, and Cu electrodes, and to understand the potential role of water in facilitating the transfer of adsorbed H atoms, we investigated the reaction kinetics of the most endergonic step (*AP → *APH·). For Cu, we used the diffused 0.75 ML H coverage, which was previously shown to lower the ΔG_*APH·_. We modeled the corresponding TS structures that connect the adsorbed *AP and *APH· intermediates in the presence of two explicit water molecules. The overall *ΔG*^*‡*^ values, calculated relative to the electrode resting state (denoted as * in Figure 4C), are consistent with ECH activity under ambient conditions: Au (+0.48 eV) < Ag (+0.73 eV) ∼ Cu (+0.76 eV).

It is noteworthy that the *ΔG*^*‡*^ barriers relative to the adsorbed *AP (Au: 0.28 eV, Ag: 0.54 eV, Cu: 0.62 eV) align with both the thermodynamic driving force for ECH and the *ΔG*_*H*_ values for a single H atom adsorbed on the different metals at 0 V vs RHE. However, these descriptors alone do not fully account for the experimental trends in *J*_*1-PEA*_ observed at –0.48 V vs RHE (Cu > Ag ∼ Au, Figure 3D). In this context, it is important to recognize that AP binding is more favorable than water binding on Ag and Au (ΔG_*H2O_ – ΔG_*AP_ = +0.17 and +0.10 eV, respectively) compared to Cu (ΔG_*H2O_ – ΔG_*AP_ = −0.05 eV). This suggests that a higher surface coverage by the organic substrate on Ag and Au can impede water adsorption and dissociation, thereby limiting the availability of surface hydrogen for ECH.

We also note that the rate of AP reduction depends not only on the value of *ΔG*^*‡*^ but also on the surface concentration of *H. Thus, the lower ECH activity observed on Ag and Au can be attributed to their predicted low hydrogen coverage at −0.48 V vs RHE. Notably, these two surfaces are not expected to develop a moderate hydrogen coverage until more negative potentials. This highlights the key role of the competition between AP and water for binding sites in governing ECH performance on Ag and Au, where efficient catalysis relies on sufficient surface hydrogen availability.

These mechanistic investigations across different metal surfaces confirm our initial hypothesis regarding the requirements for promoting ECH in alkaline conditions. Specifically, ECH catalysts need to effectively promote water dissociation in the presence of the organic substrate, subsequently reducing the generated protons to H atoms and retaining them on the surface. In this context, the thermodynamic driving force for ECH, reflected in the energy of *1-PEA (Figure 4C), correlates with the hydrogen binding strength. Moderate *ΔG*_*H*_ values are particularly beneficial in facilitating the ECH process, while favorable water binding in the presence of the organic substrate is essential to maintain an adequate hydrogen coverage on the cathode. Additionally, our findings indicate that surface H diffusion can enhance the binding of key reaction intermediates, underscoring the importance of the H surface coverage and its dynamics in ECH.

#### Rationalizing ECH Selectivity

In this section, we discuss the ECH selectivity on various cathodes, using the experimental HER activity recorded on Cu as an example (Figure S6), and the surface coverage analysis presented in Figure 2. Notably, the HER activity on Cu decreases between −0.2 and −0.4 V vs RHE, reaches a minimum between −0.4 and −0.5 V vs RHE, and then increases again up to −0.7 V vs RHE. This trend can be explained by the electroreduction of AP, which is most effective between −0.4 and −0.5 V vs RHE (Figure 3B), thereby depleting hydrogen atoms from the surface and suppressing HER. This is further supported by the coverage analysis on Cu(111) (Figure 2), which indicates that around −0.50 V vs RHE, all the *fcc* sites are already covered by H atoms and their *ΔG*_*H*_ becomes more exergonic at more negative potentials.

The increase in HER between −0.5 and −0.7 V vs RHE is attributed to the enhanced availability of weakly bound neighboring hydrogens, which can participate in the Tafel mechanism due to the coexistence of higher-density coverage states.^63^ Importantly, this relationship aligns with the central aim of our work, which is to tailor H coverages to optimize ECH activity and selectivity. Additionally, the flat adsorption geometry of AP on the metal surfaces may further inhibit the access of water molecules to the cathode, potentially limiting the contribution of the Heyrovsky step. This steric effect, combined with the relationship between high H surface coverage densities and the Volver–Tafel mechanism, led us to focus on the Volmer–Tafel pathway as the predominant HER pathway under the studied conditions. In this context, the thickest H coverage observed on Pt(111) under reaction conditions is consistent with the poor selectivity toward AP reduction across the full range of experimentally explored potentials (Figure S10).

Similarly, the weaker *ΔG*_*H*_ of Ag(111) and Au(111) compared to Cu corresponds with their enhanced ECH activity at more cathodic potentials (Figure S10). This observation supports the necessity of hydrogen coverage on the metal electrode to promote the hydrogenation of AP. The dip in FE observed with these metals at −0.68 V vs RHE is attributed to the equilibrium between different H coverages around this potential, as indicated by the crossing of multiple surface states in Figure 2, which is reported in the literature to promote HER.^64^ Overall, the net enhancement in 1-PEA production at negative potentials on Ag and Au is accompanied by a loss of selectivity, which is due to the simultaneous increase in HER via the coexistence of multiple H coverages.

After analyzing the relationship between HER and the predicted coverages of the Ag, Au, Cu, and Pt metal electrodes, we assessed the kinetics of HER on Ag(111), Cu(111), and Pt(111) via the Tafel mechanism (see Supporting Information). We determined overall activation barriers (relative to the surface resting states) of 1.02, 0.61, and 0.62 eV on Ag(111), Cu(111), and Pt(111), respectively, which are consistent with HER activity under ambient conditions. These findings also align with experimental observations, where HER contributes to a decrease in the selectivity toward the ECH of AP to 1-PEA.

Based on the promising results described above, we envision that the hydrogen surface coverage of metal cathodes could be tailored to promote other ECH reactions, provided that the experiments are performed at pH values higher than the p*K*_a_ of the hydrogenated organic substrate so that the H atoms needed for ECH must be sourced through H_2_O dissociation on the electrode surface.

### ECH of Different Organic Substrates on the Cu Catalyst

**Figure 5:**
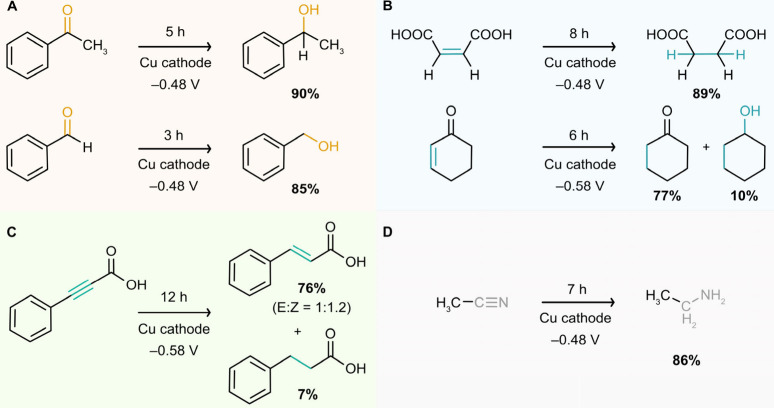
ECH of different organic functional groups on a dendritic Cu cathode. (A) ECH of the C=O group in AP (ketone) and benzaldehyde (aldehyde). (B) ECH of the C=C group in maleic acid and cyclohex-2-en-1-one. (C) ECH of the C≡C group in phenylpropiolic acid. (D) ECH of the C≡N group in acetonitrile. Yields (%) were quantified by ^1^H-NMR spectroscopy. The reaction time was optimized to achieve maximum yield of the product. All potentials are relative to the RHE.

After confirming Cu as one of the most active electrocatalysts for the ECH of AP, we expanded the substrate scope of ECH reactions that can be catalyzed by this metal (Figure 5). Following the successful ECH of a ketone (AP), we next investigated the ECH of benzaldehyde as a representative example of aldehyde hydrogenation. Under the same reaction conditions (*i*.*e*., −0.48 V vs RHE, pH = 11.8), benzaldehyde was converted into benzyl alcohol on a Cu cathode with a yield of 85% after 3 h of electrolysis (Figure 5A). Details of the ^1^H-NMR spectra and concentration profiles over time can be found in Figures S12 and S13.

Next, we explored the hydrogenation of the C=C double bond in maleic acid (MA) (Figure 5B). This process is important for upgrading biomass-derived MA into succinic acid (SA), a valuable chemical feedstock that can be used as a polymer precursor, food additive, and dietary supplement.^65,66^ The experimental conditions for the ECH of MA were the same as those for AP, except that the electrolyte pH was optimized to 7.7 instead of 11.8 (see Figure S14). The recorded ^1^H-NMR spectra of the reaction mixtures, along with the measured FE_SA_ and *J_SA_* after 1 h of electrolysis at different potentials between −0.28 and −0.68 V vs RHE, are shown in Figures S15 and S16. Similar to AP, the maximum ECH activity was observed at −0.48 V vs RHE (FE_SA_ = 75 ± 4%, *J_SA_* = 12 ± 1 mA cm^–2^). Furthermore, constant potential electrolysis at −0.48 V vs RHE resulted in 89 ± 3% conversion of MA and 89 ± 3% yield of SA after 8 h of electrolysis (Figure S16B).

The successful ECH of MA demonstrated the selective hydrogenation of C=C bonds by Cu. Therefore, we investigated the selective ECH of the C=C in an α,β-unsaturated ketone, a challenging goal in organic electrosynthesis.^12,19^ To this end, we explored the ECH of cyclohex-2-en-1-one under the same reaction conditions as for MA, except for the potential, which was optimized to −0.58 V vs RHE. After 1 h of electrolysis, 24% of the reactant was converted, yielding 22% of the desired C=C hydrogenation product (*i.e*., cyclohexanone) and only ca. 1% of the fully hydrogenated product (*i.e*., cyclohexanol; see ^1^H-NMR spectra in Figure S16).

After 6 h of electrolysis, a remarkable conversion of 88% was achieved, with a 77% yield of cyclohexanone and a 10% yield of cyclohexanol (Figure 5B and Figure S18), demonstrating the excellent chemoselectivity of this ECH process. We speculate that the extended *d*-orbitals in Cu facilitate an effective soft–soft interaction with the C=C π-bond, selectively hydrogenating the olefin in α,β-unsaturated ketones while preserving the harder carbonyl groups.

The substrate scope was further extended to the ECH of C≡C triple bonds, specifically the hydrogenation of phenylpropiolic acid (Figure 5C; NMR spectra and concentration profiles can be found in Figures S19 and S20). The selective hydrogenation of C≡C to C=C (rather than the complete hydrogenation to C–C) is a major challenge in alkyne reduction.^17^ Importantly, after 2 h of controlled potential electrolysis, 24% of the alkyne was converted into 21% of the corresponding alkene (*i.e*., cinnamic acid) and only ca. 0.5% of the alkane product (*i.e*., phenylpropanoic acid). After 12 h, a total conversion of 82% was achieved, with 76% and 7% yields of the alkene (a mixture of E/Z isomers) and alkane products, respectively. While excellent selectivities have been reported for the ECH of acetylene using LDH-derived Cu catalysts^17^ and electrochemically deposited Cu dendrites,^67^ our findings show both remarkable faradaic selectivity and chemoselectivity.

Finally, we studied the ECH of C≡N triple bonds using acetonitrile as a model substrate, as this reaction is relevant for upgrading excess acetonitrile manufacturing capacity into value-added ethylamine.^18^ Notably, acetonitrile was hydrogenated into ethylamine with an overall yield of 86% after 7 h of electrolysis under similar reaction conditions (Figure 5D, Figures S21 and S22), demonstrating the broad scope of organic functional groups that can be hydrogenated on a Cu electrocatalyst with a tailored H coverage, consistent with computational predictions.

## Conclusions

This work presents a computationally guided strategy for designing nonprecious metal catalysts tailored for the selective ECH of unsaturated organic substrates in alkaline media. By fine-tuning the density and binding strength of the hydrogen coverage on the electrode surface, we establish a series of catalyst design principles that drive this process. Specifically, we identified metal cathodes that effectively promote water dissociation, generating protons and reducing them to hydrogen atoms that are retained on the electrode surface. For optimal performance, these cathodes must exhibit balanced binding affinities for both water and the organic substrate, as well as moderate hydrogen binding strength, which is shown to increase the thermodynamic driving force toward ECH. Additionally, metals that maintain a well-dispersed hydrogen surface coverage were found to be optimal in preventing HER via a Volmer–Tafel mechanism.

DFT calculations identified Cu, Au, and Ag as promising ECH electrocatalysts. Experimental validation confirmed the superior performance of these metals for the selective ECH of acetophenone (AP) to 1-phenylethanol (1-PEA) in alkaline media, achieving FEs and yields of up to 90% at approximately –0.5 V vs RHE. Notably, these cathodes outperformed other transition metals investigated in this work, including In, Ni, and even the state-of-the-art precious metal catalyst Pt, by at least an order of magnitude in terms of FEs and yields.

The versatility of this bottom-up approach, centered on tailoring hydrogen surface coverages, was further demonstrated through the successful ECH of a wide range of unsaturated organic compounds featuring C=O, C=C, C≡C, and C≡N bonds. These reactions achieved moderate to excellent conversions and chemoselectivities (70–90%) on a Cu electrode. Beyond the hydrogen coverage, we hypothesize that the nature of the organic substrate plays an important role in the success of these ECH reactions. Specifically, the substrates examined in this work are unsaturated compounds that physisorb on the electrode surface, displacing some water molecules and competing for binding. This interaction minimizes HER while still allowing sufficient sites for H adsorption, thereby facilitating selective ECH.

Overall, this work highlights the critical role of hydrogen surface coverages in ECH and demonstrates how this knowledge can be leveraged to design high-performance ECH catalysts based on earth-abundant metals. By focusing on the interplay between hydrogen surface coverage and substrate binding, this strategy establishes a foundation for the rational design of ECH catalysts aimed at electrifying the synthesis of chemical feedstocks and value-added products. This approach has the potential to make chemical syntheses more sustainable and cost-effective, thereby contributing to the reduction of the chemical industry’s carbon footprint through the use of renewable energy sources.

## Data Availability

Computational data underlying this work, including Cartesian coordinates and energies of the optimized structures are openly accessible in the following ioChem-BD data set: https://doi.org/10.19061/iochem-bd-6-303. The experimental data can be accessed through the University of Cambridge data repository: https://doi.org/10.17863/CAM.116803.

## Supporting Information Available

Computational methods, experimental synthesis and characterization methods, as well as additional theoretical and experimental studies (PDF)
