# Supplementary material for: Driving Electrochemical Organic Hydrogenations on Metal Catalysts by Tailoring Hydrogen Surface Coverages
Source: J Am Chem Soc. 2025 Apr 8;147(16):13158–68. doi: 10.1021/jacs.4c15821 (PMC12023038; doi:10.1021/jacs.4c15821)
Supplement: Supplementary file 1 — ja4c15821_si_001.pdf [file ja4c15821_si_001.pdf]

Supporting Information

Driving electrochemical organic hydrogenations on metal catalysts by tailoring hydrogen surface coverages

Anna Ciotti,[1,†] Motiar Rahaman,[2,†] Celine Wing See Yeung,[2,†] Tengfei Li,[2,3,*] Erwin Reisner,[2,*] Max García-Melchor[1,4,5*]

[1]*School of Chemistry, CRANN and AMBER Research Centres, Trinity College Dublin, College Green, Dublin 2, Ireland*

[2]*Yusuf Hamied Department of Chemistry, University of Cambridge, Lensfield Road, Cambridge CB2 1EW, United Kingdom.*

[3]*School of Chemistry and Environment, Manchester Metropolitan University, Chester Street, Manchester M1 5GD, United Kingdom.*

[4]*Center for Cooperative Research on Alternative Energy (CIC energiGUNE), Basque Research and Technology Alliance (BRTA), Alava Technology Park, Albert Einstein 48, 01510 Vitoria-Gasteiz, Spain.*

[5]*IKERBASQUE, Basque Foundation for Science, Plaza de Euskadi 5, 48009 Bilbao, Spain.*

*Corresponding authors.

E-mails: t.li@mmu.ac.uk ; reisner@ch.cam.ac.uk ; maxgarcia@cicenergigune.com

[†]These authors contributed equally to this work.

Contents

DFT calculations 2

Electrocatalyst preparation 10

Physical characterization 11

Electrochemical measurements 11

Product quantification 11

Supplementary figures 12

Supplementary tables 24

Supplementary references 27

DFT calculations

Periodic DFT calculations were carried out with the Vienna Ab Initio Simulation Package (VASP, version 5.4.4),[1] using the Bayesian error estimation functional with van der Waals dispersions (BEEF-vdw).[2] Valence electrons were represented through plane-waves with an energy cut-off of 500 eV, while projector-augmented wave pseudopotentials[3] were used to describe the core electrons. The convergence threshold for electronic and ionic steps was set to 10^{-6} eV and 0.01 Å/eV, respectively. Gas-phase molecules were optimized at the Γ-point, while a Γ-centered k-point grid of density approximately 46 points×Å was employed for solids and the water bilayer (see details below). For slab surfaces, the same k-point density was adopted along the *x*,*y* directions, while only one k-point was taken along the *z*-axis (direction perpendicular to the surface). The electronic occupancy of the energy levels in gas-phase molecules and solids was described with a Gaussian smearing of width 0.05 eV and a Methfessel-Paxton smearing[4] of first order of width 0.2 eV, respectively. Structure relaxations were carried out with the conjugate-gradient method using a step size of 0.1 Å.

All structures were built with the Atomic Simulation Environment (ASE) software.[5] For gas-phase molecules, a vacuum of 15 Å was applied in all directions to avoid interactions between repeating images. To model bulk H_2O, a hexagonal ice-like water bilayer was constructed with six water molecules in a cell of dimensions 7.48×7.48×3.0 Å, allowing the water molecules to establish H-bonds in all directions. This bilayer was optimized relaxing the atom positions and cell volume.

For metals, the bulk structure with the lowest energy above hull was downloaded from the Materials Project database.[6] For Ag (mp-124), Au (mp-81), Cu (mp-30), Ni (mp-23), and Pt (mp-126), this corresponded to a face-centered cubic (*fcc*) cell with a lattice parameter of 4.16, 4.17, 3.62, 3.51, and 3.51 Å, respectively. On the other hand, In (mp-1055994) displayed a body-centered tetragonal cell of lattice parameters 3.31 and 5.02 Å. The lattice parameters of Ag, Au, Cu, Ni, and Pt were varied by ±1 % four times to determine the corresponding energies through constant volume calculations. Volumes and energies were then fitted to the Birch-Murnaghan equation of state,[7] and the minimum of the parabola was used to determine the equilibrium bulk structure by relaxing the atom positions and cell shape while keeping the volume constant. The optimized lattice parameters for Ag, Au, Cu, Ni and Pt were 4.22, 4.22, 3.66, 3.54, and 4.01 Å, respectively. The same procedure was adopted for In, but the lattice parameter was varied by ±1% six times to produce a good fit. This procedure led to lattice parameters of 3.33 and 5.06 Å.

To construct the surface slabs for the *fcc* metals, the above bulks were cleaved along the (111) plane (see PXRD spectrum in **Figure S2**). These slabs contain four metal layers and a vacuum of 15 Å along the direction perpendicular to the surface. For reactivity studies, *p*(6×4) supercells were employed to minimize the interaction between adsorbate species from replicated images.

To determine the most exposed facet for In, surface slabs with four atomic layers were constructed for each of the following planes: (101), (002), (110), (004), (200), (103), (211), and (202). Using the DFT-calculated surface energies for each of the slabs, a Wulff construction[8] was built with the python package WulffPack,[9] revealing that the In(101) surface is the most exposed facet.

All the metal slabs were optimized with the two bottom layers fixed to their bulk positions.

Frequency calculations were run to confirm the nature of the stationary points by vibrating all the atoms for gas-phase molecules, and only the adsorbed species for surface slabs. To this end, the finite difference method was adopted using an atomic displacement of ±0.02 Å. To further reduce computational cost, the default algorithm for the electronic loops was switched to the residual minimization method. For the calculation of the Gibbs energy corrections, any spurious frequencies below *ca.* 100 cm^{-1} were replaced by real frequencies of 12 cm^{-1} following the approach by Nørskov *et al.*[10]

Gibbs energy corrections were calculated at the experimental conditions of 298 K and 1 atm with the thermochemistry module implemented in ASE. The ideal gas model was adopted for gas-phase molecules while the harmonic model was applied to adsorbate species and the water bilayer.

Transition state (TS) structures involved in the ECH of acetophenone (AP) were modelled in the presence of explicit water molecules to mediate the hydrogen transfer from the surface coverage to the substrate, as the average distance between the closest surface H and the carbonyl group of the adsorbed AP was rather large (*i.e.* ≥3 Å). To determine the minimum number of waters needed in the TS, simulations of the ECH at the C atom of AP on the Ag(111) surface were performed by introducing up to three water molecules, as shown in **Figure S11A**. These calculations were carried out with the improved dimer method,[11] setting the convergence criterion of the ionic steps to 10^{-5} eV to reduce the computational cost. Gibbs energy corrections for the TS structures were computed as described above, vibrating the entire AP molecule, the H atoms being transferred, and the water molecules. Because the TS structure with two water molecules resulted in the lowest activation energy, $\Delta G^{\ddagger}$, (**Figure S11A**), the remaining TSs for the ECH at the carbonyl O atom on Ag, Au, and Cu were also modelled with two waters (**Figure S11B**). In this case, a preliminary nudged elastic band calculation[12] was performed between the systems featuring the adsorbed *AP and two water molecules, and the adsorbed *APH·, also in the presence of two water molecules. This nudged elastic band revealed the presence of two TSs, one pertaining to the hydrogen transfer from the surface to a water molecule, and another to the H transfer from the water to *AP. The former was higher in energy and was optimized through the improved dimer method, as described above.

Surface coverage analysis

To assess the surface coverage of the different metals, the adsorption of *H, *OH, and *O (* denotes a surface site) species was sampled on all the possible sites in a *p*(2×2) supercell for Ag, Au, Cu, Ni and Pt (*fcc*, *hcp*, *bridge* and *top*), and in a *p*(1×2) cell for In (*top*, *bridge* and *hollow*, with and without a subsurface metal atom), resulting in four sites of each type. Consequently, the addition of a single adsorbate corresponded to increasing the coverage density by less than 7%. Although larger supercells would have allowed tuning this by smaller fractions, such refinement was not expected to significantly impact the reactivity studies. The *fcc* site was the most favored for *H, *OH, and *O adsorption on all metals, except for *OH binding on Pt (more stable on a *top* site) and *O binding on In (more stable on a *bridge* site). The density of *H, *OH, and *O species was increased by sequentially populating the rest of available sites, from the most to the least favored, until either molecular species evolved during the optimization process (*e.g.* H_2) or surface reconstruction occurred.

The Gibbs binding energies of the *H, *OH, and *O species were computed at 0 V vs RHE ($\Delta G_{n*X}(0\ V_{RHE})$, X = *H, *OH and *O), using the computational hydrogen electrode model[13] and the following equations:

$$\Delta G_{n*H}(0\ V_{RHE}) = G_{n*H} - \left(E_* + {}^{n}/_{2}\, G_{H_2}\right) \quad \text{(S1)}$$

$$\Delta G_{n*OH}(0\ V_{RHE}) = G_{n*OH} - \left(E_* + nG_{H_2O} - {}^{n}/_{2}\, G_{H_2}\right) \quad \text{(S2)}$$

$$\Delta G_{n*O}(0\ V_{RHE}) = G_{n*O} - \left(E_* + nG_{H_2O} - nG_{H_2}\right) \quad \text{(S3)}$$

where G_{n*X} is the Gibbs energy of *n* adsorbed species *X, E_* is the potential energy of the bare metal surface, G_{H2} is the energy of the hydrogen molecule, and G_{H2O} the energy of a water molecule. The plots of ΔG_{n*X} against the applied potential vs RHE (U_{RHE}), reported in **Figure 2** of the main text, were derived from Eqs. S4-S6 using the following expressions:

$$\Delta G_{n*H}(V_{RHE}) = G_{n*H} - \left(E_* + {}^{n}/_{2}\, G_{H_2}\right) + nU_{RHE} \quad \text{(S4)}$$

$$\Delta G_{n*OH}(V_{RHE}) = G_{n*OH} - \left(E_* + nG_{H_2O} - {}^{n}/_{2}\, G_{H_2}\right) - nU_{RHE} \quad \text{(S5)}$$

$$\Delta G_{n*O}(V_{RHE}) = G_{n*O} - \left(E_* + nG_{H_2O} - G_{H_2}\right) - 2nU_{RHE} \quad \text{(S6)}$$

We note that solvent effects are not included in this analysis. While this omission may influence the energetics of *O and *OH adsorbates due to their ability to form hydrogen bonds with solvent molecules, the reducing conditions of our ECH studies preclude the presence of these adsorbates. Moreover, coverage studies performed at the solid-vacuum interface remain widely applicable in computational electrochemistry, given the inherent challenges of accurately modelling the solid-liquid interface with DFT.

Modelling H_2O binding

A water molecule was bound to the *p*(6×4) surface slabs of the various metals, featuring the hydrogen coverages that were used to model *AP adsorption. This corresponds to a supercell bearing two *H atoms for Ag(111) and Au(111), a 0.75 monolayer (ML) H coverage on Cu(111), and a 1.00 ML H coverage on Ni(111) and Pt(111). The water molecule was positioned to bind through an oxygen lone pair at the *bridge*, *hcp*, *top*, and *fcc* sites, as well as on a hydrogen atom of the surface coverage. The Gibbs adsorption energy of the water molecule, ΔG_{*H2O}, was then computed relative to both a water molecule in the gas-phase and a water molecule in the bilayer. For Ag(111) and Au(111), Eqs. S7a-b were used, while Eqs. S8a-b were employed for Cu(111), Ni(111), and Pt(111).

$$\Delta G_{*H_2O} = G_{*H_2O} + 2G_{corr,*H} - \left(E_* + G_{H_2O} + G_{H_2}\right) + 2eU_{RHE} \quad \text{(S7a)}$$

$$\Delta G_{*H_2O} = G_{*H_2O} + 2G_{corr,*H} - \left(E_* + \frac{1}{6}G_{bilayer} + G_{H_2}\right) + 2eU_{RHE} \quad \text{(S7b)}$$

$$\Delta G_{*H_2O} = G_{*H_2O} - \left(E_* + G_{H_2O}\right) \quad \text{(S8a)}$$

$$\Delta G_{*H_2O} = G_{*H_2O} - \left(E_* + \frac{1}{6}G_{bilayer}\right) \quad \text{(S8b)}$$

where E_* represents a *p*(6×4) surface slab featuring two *H atoms for Ag(111) and Au(111), and the hydrogen-covered surface on Cu(111), Ni(111), and Pt(111), and $G_{bilayer}$ is the Gibbs energy of the water bilayer. The ΔG_{*H2O} values relative to the water bilayer are reported in **Figure 4B** of the main text, while those computed relative to the water molecule were 0.41, 0.35, 0.16, 0.19, and 0.19 eV for Ag(111), Au(111), Cu(111), Ni(111), and Pt(111), respectively. Notably, the difference in ΔG_{*H2O} between the two references is *ca*. 0.06 eV, and the trend between water and *AP binding is maintained.

Calculation of the ECH energetics

The Gibbs reaction energies reported in this work were calculated within the framework of the computational hydrogen electrode model, relative to the AP and H_2 molecules in the gas phase and the resting state of the *p*(6×4) surface slabs predicted under experimental conditions (**Figure 4A**).

Because on Ag(111) and Au(111) this corresponded to the bare slab, the first step in the ECH mechanism involved the binding of the two hydrogens necessary for the ECH of AP to 1-PEA. Subsequently, AP was adsorbed and reduced, first to *APH· and then to *1-PEA, which desorbed to restore the bare catalyst surface. All the energies were computed as follows:

$$\Delta G_{2*H} = G_{2*H} - \left(E_* + G_{H_2}\right) + 2eU_{RHE} \quad \text{(S9)}$$

$$\Delta G_{*AP} = G_{*AP} + 2G_{corr,*H} - \left(G_{AP} + E_* + G_{H_2}\right) + 2eU_{RHE} \quad \text{(S10)}$$

$$\Delta G_{*APH\cdot} = G_{*APH\cdot} + G_{corr,*H} - \left(G_{AP} + E_* + G_{H_2}\right) + 2eU_{RHE} \quad \text{(S11)}$$

$$\Delta G_{*1-PEA} = G_{*1-PEA} - \left(G_{AP} + E_{*} + G_{H_2}\right) + 2eU_{RHE} \tag{S12}$$

$$\Delta G_{1-PEA} = G_{1-PEA} - \left(G_{AP} + E_{*} + G_{H_2}\right) + 2eU_{RHE} \tag{S13}$$

where G_{*X} is the Gibbs energy of the adsorbed species *X, G_X the Gibbs energy of the same species in the gas phase, E_* is the energy of the bare metal surface, $G_{corr,*H}$ the vibrational contribution to the energy of an adsorbed *H on the surface coverage, and e is the charge of the electron.

On the other hand, the resting state of Cu(111), Ni(111), and Pt(111) was predicted to feature a hydrogen coverage. Thus, the ECH energetics were calculated relative to their corresponding H-covered p(6×4) slabs. The ECH mechanism on these surfaces involved AP adsorption, followed by two proton-coupled steps to yield *APH· and *1-PEA, and the refilling of the two hydrogen vacancies generated during the process. The Gibbs energies of these elementary steps were computed as:

$$\Delta G_{*AP} = G_{*AP} - \left(G_{AP} + E_{*}\right) \tag{S14}$$

$$\Delta G_{*APH\cdot} = G_{*APH\cdot} - \left(G_{AP} + E_{*} + G_{corr,*H}\right) \tag{S15}$$

$$\Delta G_{*1-PEA} = G_{*1-PEA} - \left(G_{AP} + E_{*} + 2G_{corr,*H}\right) \tag{S16}$$

$$\Delta G_{2*H} = G_{2*H} - \left(G_{AP} + E_{*} + G_{H_2}\right) + 2eU_{RHE} \tag{S17}$$

$$\Delta G_{1-PEA} = G_{1-PEA} - \left(G_{AP} + E_{*} + G_{H_2}\right) + 2eU_{RHE} \tag{S18}$$

For the calculation of the activation barriers ($\Delta G^{\ddagger}$) in the ECH of AP, it is important to correctly account for the role of solvent molecules. Using a gas-phase water molecule as a reference for the solvent would introduce a fictitious bias by increasing the number of hydrogen bonds in the TS. Conversely, referencing the TS energy to a water bilayer would reduce the number of hydrogen bonds, potentially underestimating the solvent effect. To address this, $\Delta G^{\ddagger}$ values were computed relative to the system containing the adsorbed *AP and the appropriate number of water molecules introduced to facilitate hydrogen transfer ($G_{*AP+n*H2O}$). Given that *AP binding was endergonic on all metals, the overall $\Delta G^{\ddagger}$ was calculated by incorporating ΔG_{*AP} into the calculation:

$$\Delta G^{\ddagger} = G^{\ddagger} - \left(G_{*AP+n*H_2O} + G_{corr,*H}\right) + \Delta G_{*AP} \tag{S19}$$

where $G^{\ddagger}$ is the Gibbs energy of the TS structure and n represents the number of water molecules involved in the hydrogenation step.

*<u>Modelling ECH of *AP at the carbonyl C atom</u>*

For all metals, we also explored the possibility of hydrogenating the carbonyl carbon of *AP first, rather than the oxygen atom (as reported in the main text). For Ag and Cu, the ECH of *AP at the carbon was found to be thermodynamically more favorable than the ECH at the oxygen (+0.13 vs

+0.68 eV, and +0.41 vs +0.49 eV, respectively), with the corresponding pathway shown in **Figure S11C**. Conversely, for Au, Ni, and Pt, ECH at the carbon was less favored than at the oxygen, with $\Delta G_{*APH\cdot}$ values of +0.60 eV (vs +0.28 eV), +1.79 eV (vs +0.79 eV) and +1.46 eV (vs +0.52 eV), respectively.

While the *APH· intermediate hydrogenated at the carbon was stabilized through a metal-oxygen bond with the electrode surface, the *APH· intermediate hydrogenated at the oxygen was stabilized by the delocalization of its radical character over the aromatic ring, as discussed in the main text. This was confirmed by inspecting the magnetic moments, which, in the case of Cu, indicated a radical character of 0.47 e on the oxygen, 0.19 e on the carbonyl carbon, 0.03 e on the phenyl carbon bearing the carbonyl group, 0.05 e at the ortho positions, 0.02 e at the meta positions, and 0.06 at the para position.

*Modelling the ECH of AP on dynamic *H surface coverages*

As discussed in the main text and shown in **Figure S11E**, the diffusion of surface H atoms can lower the energy required for the ECH of *AP at the oxygen atom on the Cu(111). Consequently, the ECH at the carbon atom was reassessed under these dynamic conditions, revealing that ECH at the carbon atom becomes thermodynamically favored when surface H atoms are allowed to diffuse. This finding underscores the importance of considering both the surface coverage under relevant reaction conditions and the dynamics of H atoms on the surface. The complete energy profile for ECH on Cu(111) is presented in **Figure S11C**.

Following this, the ECH of *AP at the oxygen atom was investigated with 0.50 ML H and 1.00 ML H coverages on Cu(111), as these coverages are predicted to have energies similar to the most stable 0.75 ML H coverage (see **Figure 2**). The calculated $\Delta G_{*APH\cdot}$ values for these coverages were +0.54 eV and +0.39 eV, respectively, which aligns with the more favorable ΔG_H at lower coverages (–0.22 eV and –0.13 eV for 0.50 and 1.00 ML H coverages, respectively), as shown in **Figure S11E**.

Finally, H diffusion was examined on the 0.50 ML H coverage of Cu(111) to determine whether $\Delta G_{*APH\cdot}$ could be further lowered by displacing the surface H atoms away from the *APH· intermediate (see main text). Due to the consistent Gibbs energy corrections observed for the *APH· intermediate across all metals, the same Gibbs contributions calculated for the uniform 0.75 ML H coverage were applied to the *APH· intermediates in the presence of a diffused H coverage.

Calculation of gas-phase errors in the ECH of AP

To account for potential inaccuracies in the adsorption energies of AP and 1-PEA calculated using DFT, gas-phase errors were estimated as the difference between their theoretical and experimental formation enthalpies ($\Delta H^{\circ}_{f,theo}$ and $\Delta H^{\circ}_{f,exp}$, respectively).[14] Since O_2 appears as a reactant in the formation of both AP and 1-PEA, its energy was also corrected based on the formation enthalpy of H_2O. The enthalpies of H_2 and a graphene monolayer with a two-atom unit cell (used as a reference for C; Materials Project ID: mp-48), were assumed to be accurate.[14]

The $\Delta H^{\circ}_{f,theo}$ values of AP, 1-PEA, and H_2O were calculated as follows, using the data summarized in **Table S2**:

$$\Delta H^{\circ}_{f,theo}(AP) = H_{AP} - \left(4H_{H_2} + 8H_C + \frac{1}{2}H_{O_2}\right) = 0.40 \text{ eV} \qquad (S20)$$

$$\Delta H^{\circ}_{f,theo}(1-PEA) = H_{1-PEA} - \left(5H_{H_2} + 8H_C + \frac{1}{2}H_{O_2}\right) = 0.25 \text{ eV} \qquad (S21)$$

$$\Delta H^{\circ}_{f,theo}(H_2O) = H_{H_2O} - \left(H_{H_2} + \frac{1}{2}H_{O_2}\right) = -2.13 \text{ eV} \qquad (S22)$$

where H_X represents the enthalpy of species X. **Table S2** lists the electronic energy and enthalpy contributions for H_2, O_2, H_2O, AP, 1-PEA, and the two-atom unit cell of the graphene monolayer, which was divided by two to obtain H_C. The enthalpy contributions were calculated by summing the zero-point energy and the integrated heat capacity at constant pressure over the 0-298 K temperature range.

The $\Delta H^{\circ}_{f,exp}$ of AP, 1-PEA and H_2O were taken from experimental databases:[15–17] –0.899, –1.341, and –2.506 eV, respectively. The error in the O_2 energy was then calculated as the difference between the theoretical and experimental formation enthalpies of H_2O:[18]

$$Error_{O_2} = 2 \times \left(\Delta H^{\circ}_{f,exp}(H_2O) - \Delta H^{\circ}_{f,theo}(H_2O)\right) = -0.75 \text{ eV} \qquad (S23)$$

This result is consistent with the value of –0.81 eV reported in the literature for the BEEF-vdw functional.[14] Subsequently, corrections to the DFT energies of AP and 1-PEA were calculated as follows, including half of the O_2 error term to account for its role as a reactant in Eqs. S20-S21:

$$Error_{AP} = \Delta H^{\circ}_{f,theo}(AP) - \Delta H^{\circ}_{f,exp}(AP) + \frac{1}{2}Error_{O_2} = 0.92 \text{ eV} \qquad (S24)$$

$$Error_{1-PEA} = \Delta H^{\circ}_{f,theo}(1-PEA) - \Delta H^{\circ}_{f,exp}(1-PEA) + \frac{1}{2}Error_{O_2} = 1.22 \text{ eV} \qquad (S25)$$

These results indicate that DFT energies for AP and 1-PEA are overestimated by *ca.* 1 eV. Consequently, the energy difference between 1-PEA and AP should be larger by the difference in

their respective gas-phase errors, which amounts to –0.29 eV. This adjustment is represented with a dashed line in the reaction profiles shown in **Figure 4** and **Figure S11**.

Notably, incorporating the correction for AP into the reaction pathway would render all intermediates more endergonic by +0.92 eV. However, this significant increase in the energy profile is inconsistent with the ECH activity observed in experiments and the experimentally reported adsorption of AP on a Pt(111) electrode.[19] This discrepancy likely arises because adsorbate energies are not corrected in this framework. For this reason, only the overall reaction energy was corrected.

Kinetic investigation of Tafel HER

HER was modelled via the Tafel mechanism on Ag(111), Cu(111) and Pt(111). Similar results were envisioned between Ag(111) and Au(111), and Pt(111) and Ni(111), in light of their similar ΔG_H and coverage density. Because the Tafel mechanism only involves two adsorbed H atoms, Cu(111) and Pt(111) *p*(2×2) supercells were adopted in their respective coverage predicted under experimental conditions. Since the Ag(111) surface was instead expected to be bare, the covered state of lowest energy was employed for the HER study, *i.e.* the 0.25 H. To accommodate two H atoms, an Ag(111) *p*(4×2) supercell was therefore adopted.

To identify the TS of Tafel HER via a nudged elastic band investigation, the coverage configurations featuring two neighboring H atoms were first sampled, as well as H_2 adsorption on *bridge*, *fcc*, *hcp*, and *top* sites. Given the preference for hydrogen binding on the *fcc* site, one of the reactive H atoms was envisioned to occupy such position, while the second was displaced in a vicinal *bridge*, *fcc*, *hcp* and *top*. To prevent H migration to the *fcc* sites in the sampling of the H-H configurations, and H_2 desorption in the modelling of its binding, the quasi-Newton algorithm was used with a finite step size of 0.05 Å.

On Ag(111) and Cu(111), H_2 was not stable and desorbed from all positions but the *bridge*. Furthermore, the latter featured an imaginary frequency in the direction of hydrogen coupling. Thus, searching the TS via a nudged elastic band calculation was not viable. Instead, an improved dimer calculation was conducted on the structure featuring H_2 on the *bridge* site, with the settings described above. Conversely, the sampling of the H-H states on Pt(111) indicated the *fcc* + *fcc* and *fcc* + *top* configurations as stable under the computational settings here employed. Thus, they were adopted as initial states for the nudged elastic band. The *top* site was instead chosen for the final state, since it was the preferred position for H_2 binding. Both nudged elastic band searches were followed by an improved dimer optimization with the settings outlined above, converging to the same stationary point. Lastly, the activation barriers $\Delta G^{\ddagger}_{HER}$ were computed on Ag(111) with respect to the bare surface (Eq. S26), and on Cu(111) and Pt(111) with respect to the covered slabs (Eq. S27):

$$\Delta G^{\ddagger}_{HER} = G^{\ddagger} - \left(E_* + G_{H_2}\right) + 2eU_{RHE} \quad (S26)$$

$$\Delta G^{\ddagger}_{HER} = G^{\ddagger} - \left(E_* + 2G_{corr,*H}\right) \quad (S27)$$

Electrocatalyst preparation

Substrate preparation for metal electrocatalyst fabrication

The metal electrocatalysts were electrodeposited on Cu foil substrates. A Cu foil (10 cm × 10 cm, 99.9% pure, Alfa Aesar) was cut into pieces with 0.7 cm × 3 cm dimensions and cleaned by sonicating in absolute ethanol for 15 min. The cleaned foils were masked with Teflon to fix an active area of 0.7 cm × 1.2 cm (0.84 cm^2) for catalyst fabrication.

Cu catalyst electrodeposition

The dendritic metal catalysts were synthesized by a dynamic hydrogen bubble templated assisted electrodeposition method following a previously reported protocol.[20] A three-electrode set-up was used for the electrodeposition where a double junction Ag/AgCl (saturated NaCl) electrode was used as a reference, a Pt foil as a counter, and a Teflon masked Cu foil as a working electrode. For Cu catalyst fabrication, 0.05 M $CuSO_4 \cdot 5H_2O$ in 1.5 M H_2SO_4 electrolyte was used. A galvanostatic electrodeposition was carried out by applying −3 A cm^{-2} current density for 40 seconds. Vigorous H_2 bubble formation occurred in the strongly acidic electrolyte medium under cathodic conditions, which acted as a template to form metallic Cu catalysts from Cu^{2+} ions. After electrodeposition, the catalysts were washed with Milli-Q water to remove excess electrolytes and dried under N_2 flow. Two other Cu catalysts were prepared by changing the deposition time to 10 and 80 seconds.

Ag catalyst electrodeposition

The Ag catalysts were electrodeposited using a 0.02 M Ag_2SO_4 in 1.5 M H_2SO_4 electrolyte. A galvanostatic current density of −3 A cm^{-2} was applied for 20 seconds to form Ag catalysts. After electrodeposition, Ag catalysts were washed with Milli-Q water and dried under N_2 flow.

Au catalyst electrodeposition

0.01 M $HAuCl_4$ in 1.5 M H_2SO_4 electrolyte was used for Au catalyst preparation. Galvanostatic electrodeposition was carried out by applying −3 A cm^{-2} for 30 seconds. After preparation, Au catalysts were washed with Milli-Q water and dried under N_2 flow.

In catalyst electrodeposition

The In catalyst was electrodeposited using 0.02 M $In_2(SO_4)_3 \cdot xH_2O$ in 1.5 M H_2SO_4 electrolyte. A −3 A cm^{-2} current density was applied for 30 seconds to electrodeposit In catalysts. In catalysts were washed in Milli-Q water and dried under N_2 flow.

Pt and Ni electrocatalysts

Commercial Pt foil (99.9%, Alfa Aesar) and Ni foam (99.9%, 1.6 mm thickness, MJ group) were also used as electrocatalysts.

Physical characterization

A TESCAN MIRA3 field emission gun-scanning electron microscope was used for the SEM analysis. PXRD measurement with Cu catalyst was performed by a Panalytical X'Pert Pro (K alpha Cu radiation) diffractometer with a scan rate 1° min^{-1}.

Electrochemical measurements

Electrochemistry experiments were performed in a gas-tight two-compartment electrochemical cell separated by a proton exchange membrane (Nafion[117]) at room temperature. The cathodic compartment contained 9 mL of water, 1 mL of ethanol (to increase the solubility of organic compounds), 0.25 M KH_2PO_4/KOH buffer electrolyte (pH 11.8) and 0.05 M organic substrate. The electrocatalyst (surface area = 0.84 cm^2) was used as working electrode. A Pt mesh counter electrode in the anodic compartment was immersed in 5 mL of 0.25 M KH_2PO_4/KOH aqueous solution (pH = 11.8). All potentials were measured against an Ag/AgCl (saturated NaCl) reference electrode (BASi MW-2030) stored in saturated NaCl and converted to the RHE reference scale using the relation:

$$E(V_{RHE}) = E(V_{Ag/AgCl}) + 0.197\,V + 0.059 \times pH \qquad (S22)$$

The electrochemical hydrogenation (ECH) experiments were performed at constant potential and the reported Faradaic efficiencies (FE_x) and partial current densities (J_x) towards a certain product were determined according to the following equations:

$$FE_x = \frac{10\,mL * conc._x\left(\frac{mol}{mL}\right) * n * 96485}{charge\ (C)} * 100\% \qquad (S23)$$

$$J_x = J_{total} * FE_x \qquad (S24)$$

Product quantification

The reaction aliquots were identified and quantified by ^{1}H-NMR spectroscopy. The NMR data were collected using a Bruker Neo Prodigy 400 MHz NMR spectrometer. Deuterium oxide (D_2O) was added into the aqueous reaction mixture as the deuterated solvent. Benzene-1,3,5-tricarboxylic acid was used as the internal standard for quantification. A series of standard solutions with known concentrations of compounds were prepared to plot a linear calibration curve.

The gas products were analyzed by injecting 50 μL of gas from the headspace of the cathode compartment into a Shimadzu Tracera 2010 gas chromatograph equipped with a RT-Molsieve 5A (30 m × 0.53 mm ID, Restek) column. The H_2 was detected by a thermal conductivity detector (TCD) using helium as the carrier gas. Calibration curves for H_2 were created by plotting the peak area versus concentration in standard calibration gas.

Supplementary figures

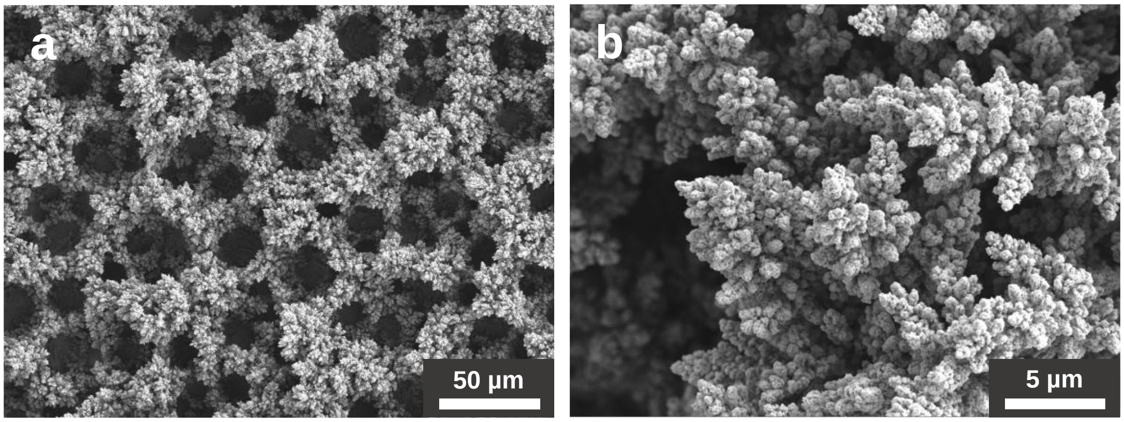

Figure S1. Low (a) and high-resolution (b) SEM analyses of a Cu electrocatalyst (40 seconds deposition time) showing the dendritic microporous morphology.

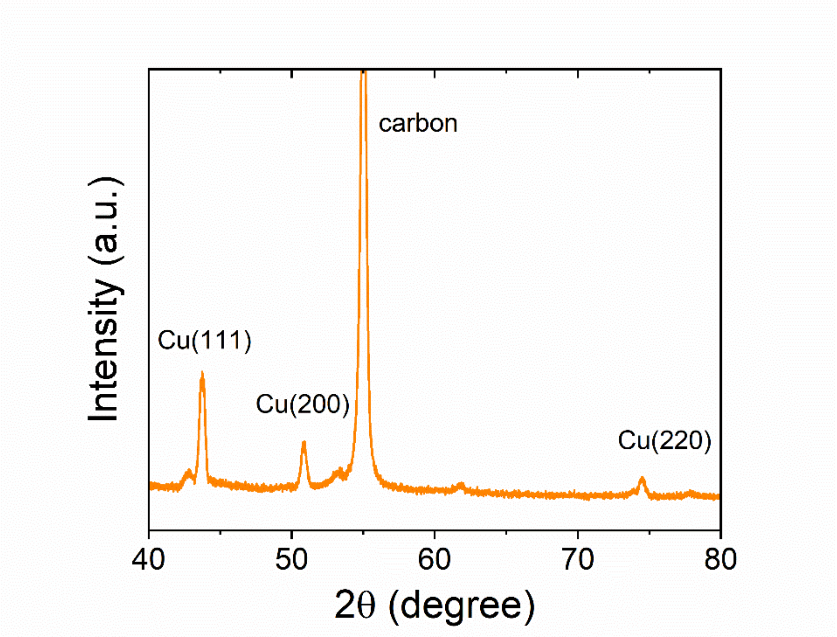

Figure S2. XRD analysis of a Cu catalyst deposited (40 seconds) on a carbon paper.

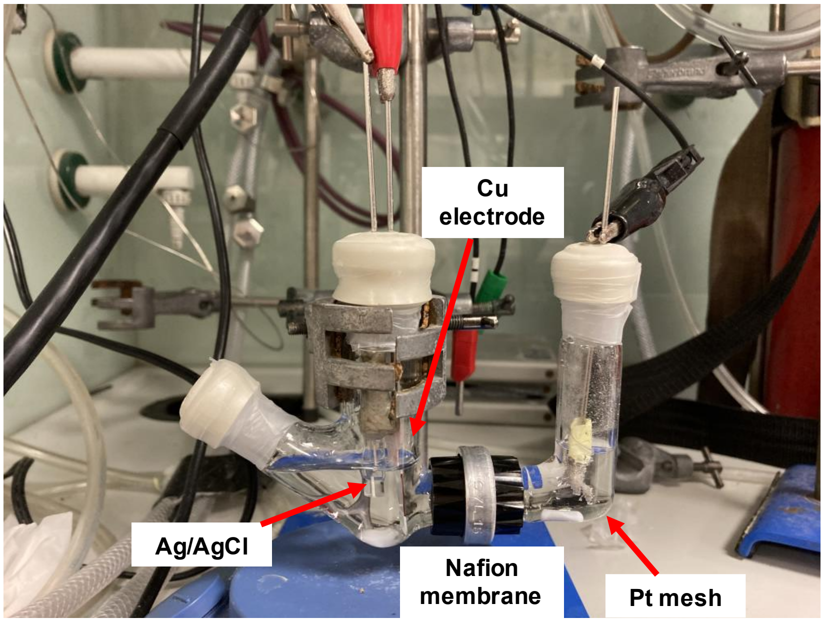

Figure S3. Electrochemical cell used to drive the ECH of organic substrates.

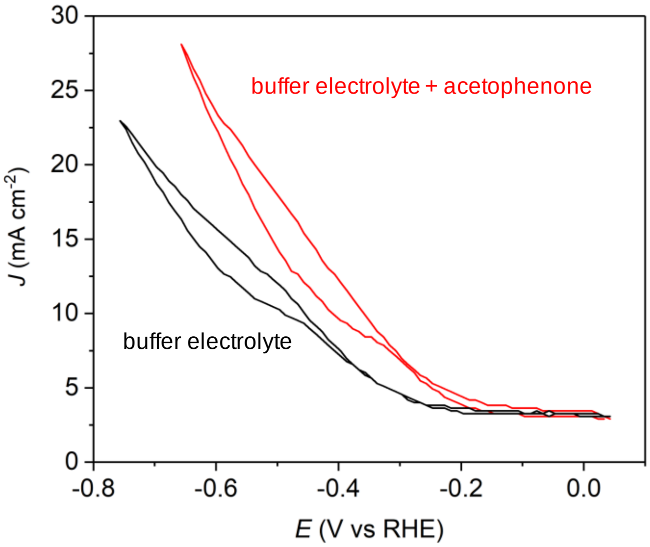

Figure S4. Cyclic voltammograms measured on a Cu electrocatalyst before and after adding 0.5 mmol of acetophenone into the electrolyte. Experimental conditions: Cu cathode (surface area = 0.84 cm^2) immersed in 10 mL of aqueous solution containing 10 vol% ethanol and 0.25 M KH_2PO_4/KOH buffer electrolyte (pH = 11.8). Potentials were measured against an Ag/AgCl reference electrode and reported versus RHE.

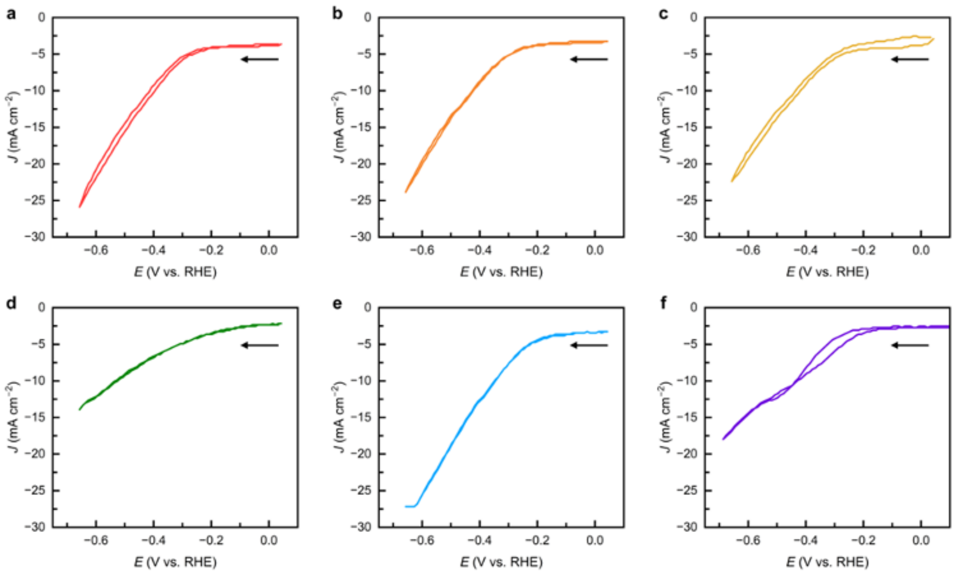

Figure S5. Cyclic voltammograms of various electrocatalysts for AP hydrogenation. (a) Cu, (b) Au, (c) Ag, (d) Pt, (e) Ni, and (f) In. The black arrows denote the initial scanning direction. Conditions: 0.5 mmol of AP, 10 mL of aqueous solution containing 10 vol% ethanol and 0.25 M KH_2PO_4/KOH buffer electrolyte (pH = 11.8), scan rate: 25 mV s^{-1}, surface area of electrocatalyst: 0.84 cm^2.

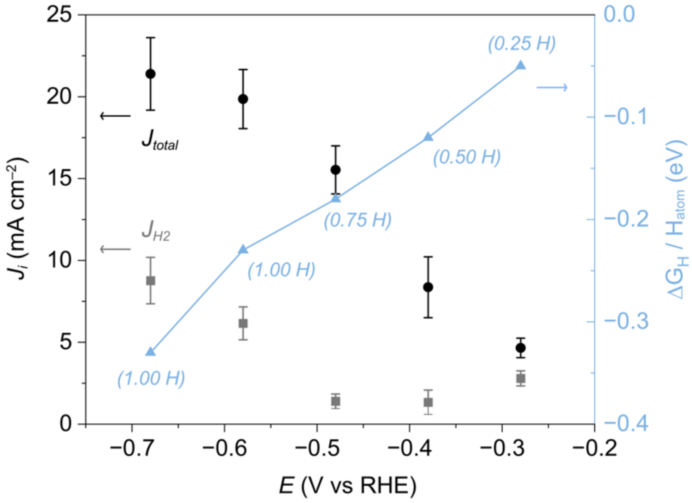

Figure S6. Total current density (J_{total}) and partial current density for H_2 (J_{H2}) during AP hydrogenation on a Cu electrocatalyst at various potentials. The calculated Gibbs energies for hydrogen adsorption per H atom (ΔG_H/Hatom) on the lowest energy surface coverages at each potential, as predicted in **Figure 2**, are also shown. The hydrogen coverage corresponding to each applied potential is provided in parenthesis.

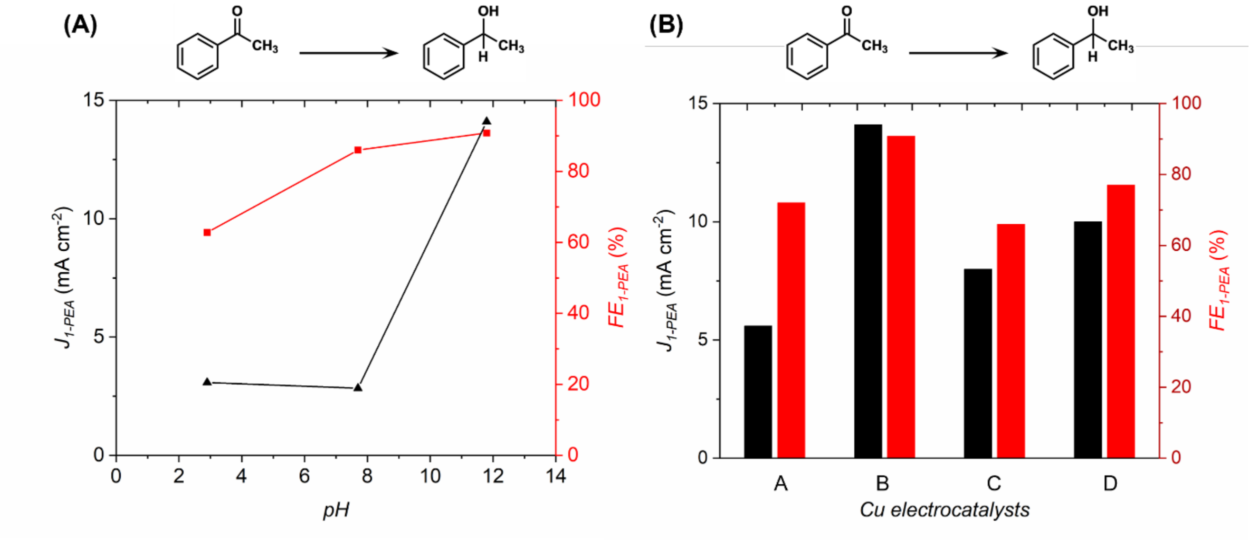

Figure S7. Optimization of electrolyte pH and Cu electrocatalysts for the ECH of AP to 1-PEA. $FE_{1\text{-}PEA}$ and $J_{1\text{-}PEA}$ were measured after 1 hour of electrolysis at –0.48 V vs RHE. (**A**) Optimization of the electrolyte pH (pH = 11.8, 7.7, and 2.9). (**B**) Optimization of different Cu electrocatalysts at pH = 11.8. Electrode A: Cu prepared by electrodeposition of 10 seconds. Electrode B: Cu prepared by electrodeposition of 40 seconds. Electrode C: Cu prepared by electrodeposition of 80 seconds. Electrode D: oxide-derived Cu.

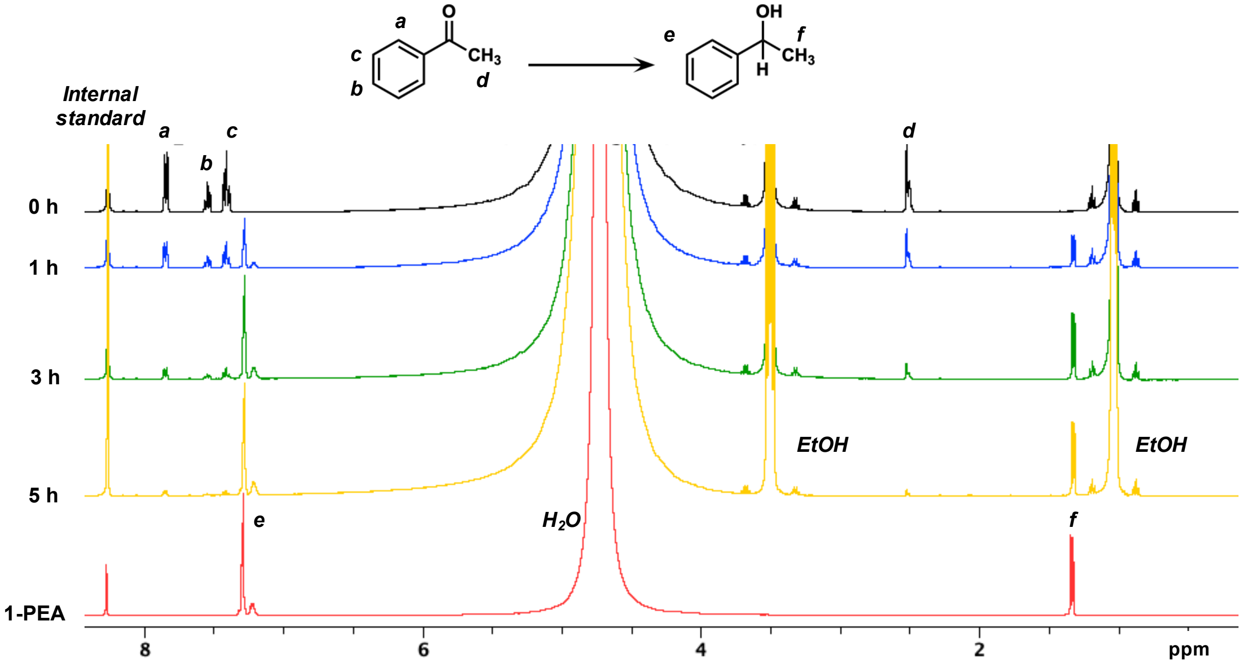

Figure S8. Crude ^{1}H-NMR spectra (64 scans) for the reaction mixtures obtained in the ECH of AP after 0, 1, 3, and 5 hours of electrolysis. D_2O was used as the deuterated reagent for the ^{1}H-NMR measurement. Benzene-1,3,5-tricarboxylic acid (singlet, δ = 8.3 ppm) was added as an internal standard to quantify the amount of reactant and product.

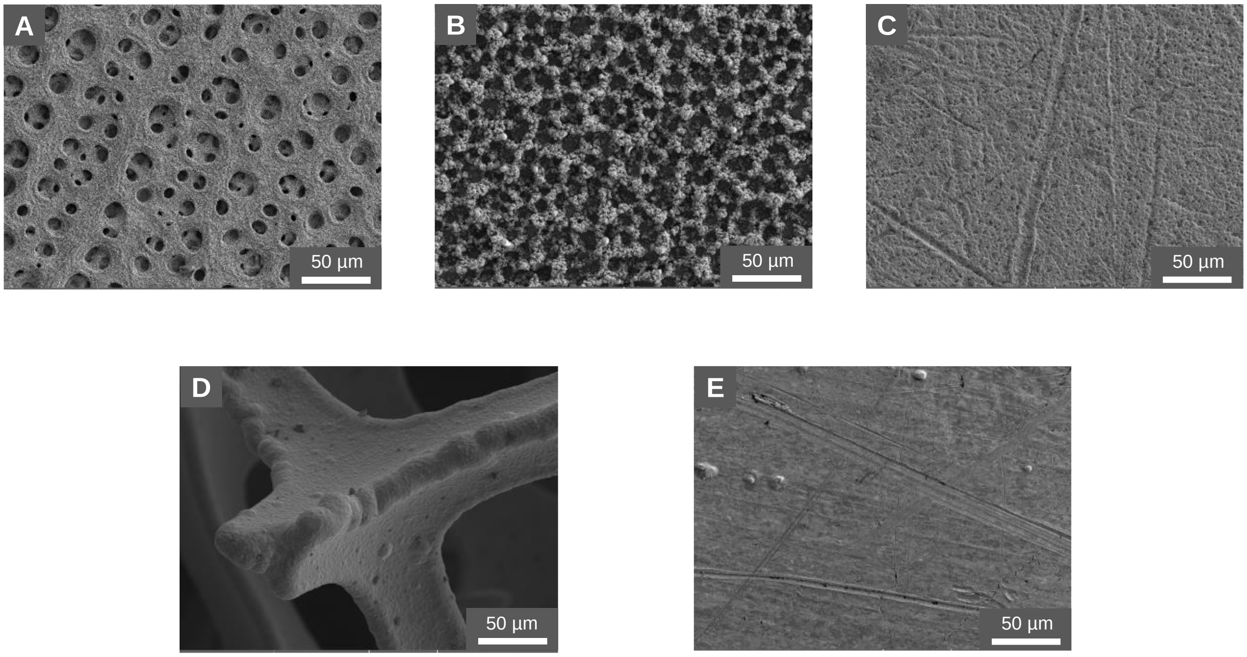

Figure S9. SEM images of electrodeposited (A) Ag, (B) Au, (c) In catalysts, and commercial (D) Ni mesh and (E) Pt foil catalysts.

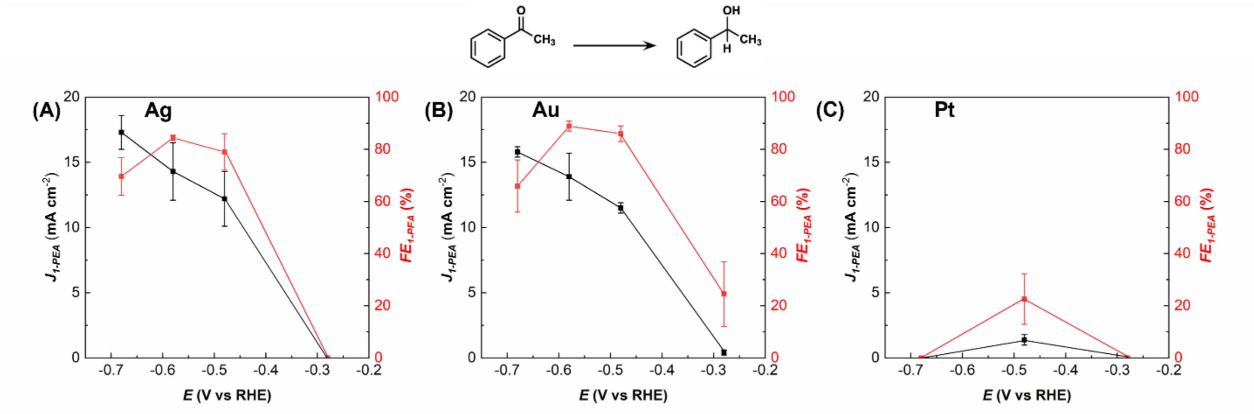

Figure S10. Optimization of the applied potential on different metal catalysts. $J_{1\text{-PEA}}$ (black) and $FE_{1\text{-PEA}}$ (red) for the ECH of AP to 1-PEA were measured on Ag (**A**), Au (**B**), and Pt (**C**) at potentials between –0.28 and –0.68 V vs RHE after 1-hour electrolysis. Error bars correspond to the standard deviation of triplicate (n = 3) experiments.

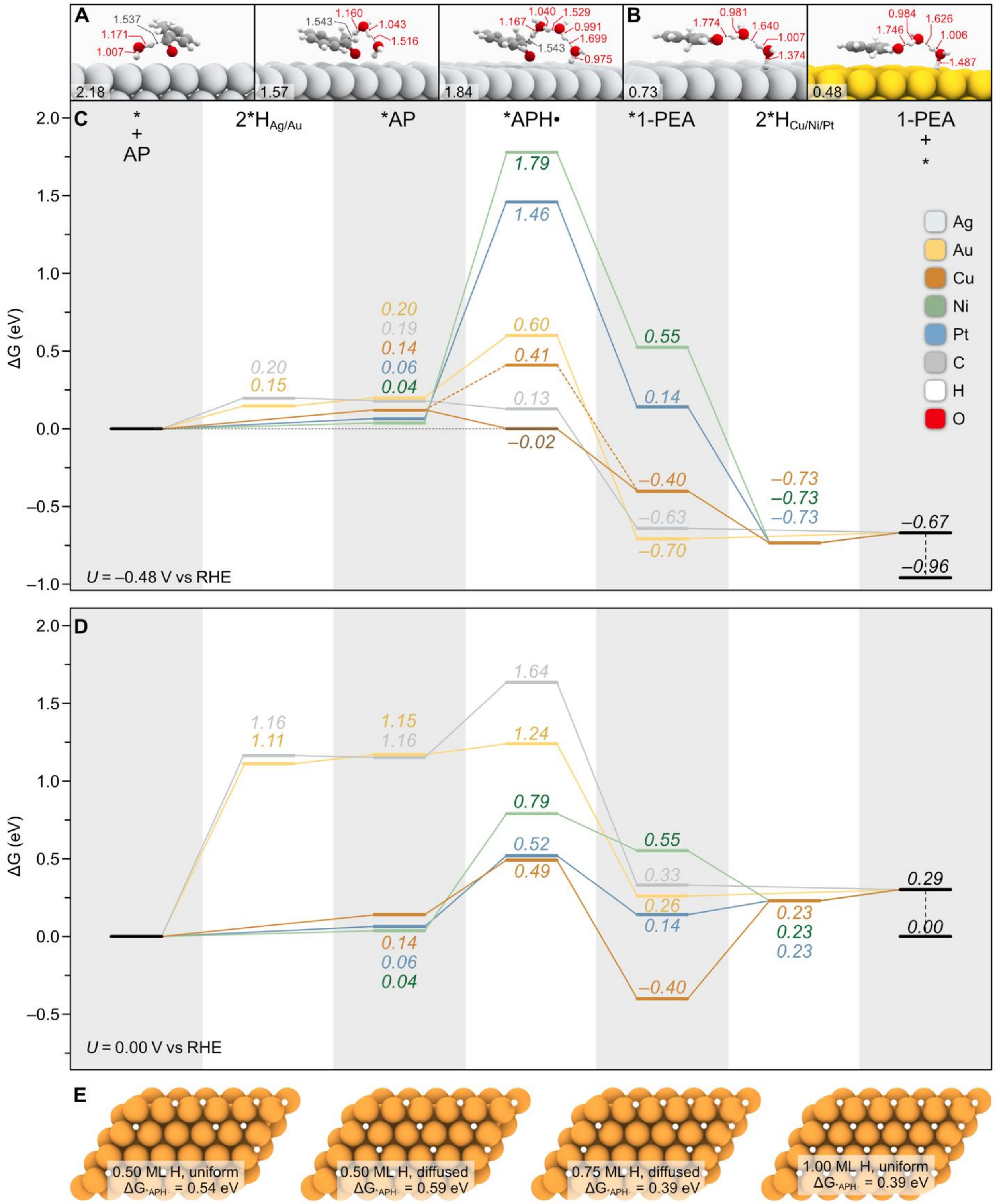

Figure S11. (**A**) Optimized TS structures for the ECH of *AP at the carbonyl C atom on the *p*(6×4)-Ag(111) surface with two adsorbed hydrogen atoms assisted by one, two, and three water molecules. Relevant bond distances (in Å) involving C and O atoms are shown in grey and red, respectively. (**B**) Optimized TS structures for the ECH of *AP at the carbonyl O atom on *p*(6×4)

supercells of Ag(111) and Au(111) with two adsorbed *H atoms assisted by two water molecules. Corresponding $\Delta G^{\ddagger}$ values (in eV) are provided for each TS structure, along with relevant distances in Å. (**C**) Gibbs energy profiles for the ECH of *AP to 1-PEA calculated at –0.48 V on *p*(6×4) supercells of Ag(111) and Cu(111) on *p*(6×4) supercells of Ag(111) and Cu(111) with two adsorbed *H atoms and a 0.75 ML H coverage, respectively. The $\Delta G_{*APH\cdot}$ value for the diffused 0.75 ML H coverage on Cu(111) is highlighted in dark brown. (**D**) Gibbs energy profile for the ECH of AP to 1-PEA calculated at 0.00 V vs RHE. On Ag(111) and Au(111), the ECH mechanism involves the reduction of two protons ($2{*}H_{Ag/Au}$), AP adsorption (*AP), hydrogenation at the O atom (*APH·), hydrogenation at the C atom (*1-PEA), and product desorption (1-PEA). On Cu(111), Ni(111), and Pt(111), the ECH mechanism involves AP adsorption (*AP), hydrogenation at the O (*APH·), hydrogenation at the C (*1-PEA), refilling of the H vacancies ($2{*}H_{Cu/Ni/Pt}$), and product desorption (1-PEA). The energy of 1-PEA corrected by its estimated gas-phase error with respect to AP is also shown with a dotted line. (**E**) Top view representations of *p*(6×4)-Cu(111) supercells illustrating, from left to right, a uniform 0.50 ML H coverage, a diffused 0.50 ML H coverage, a diffused 0.75 ML H coverage, and a 1.00 ML H coverage. The corresponding $\Delta G_{*APH\cdot}$ values are indicated for each configuration. We note that the calculated $\Delta G_{*APH\cdot}$ values for diffused 0.50 ML H and 0.75 ML H coverages, uniform 0.50 ML H and 1.00 ML H coverages, and the uniform 0.75 ML H coverage used in **Figure 4** fall within a ±0.1 eV range. While this variation is unlikely to alter the conclusions of this work, it highlights the potential significance of hydrogen surface coverage dynamics in ECH.

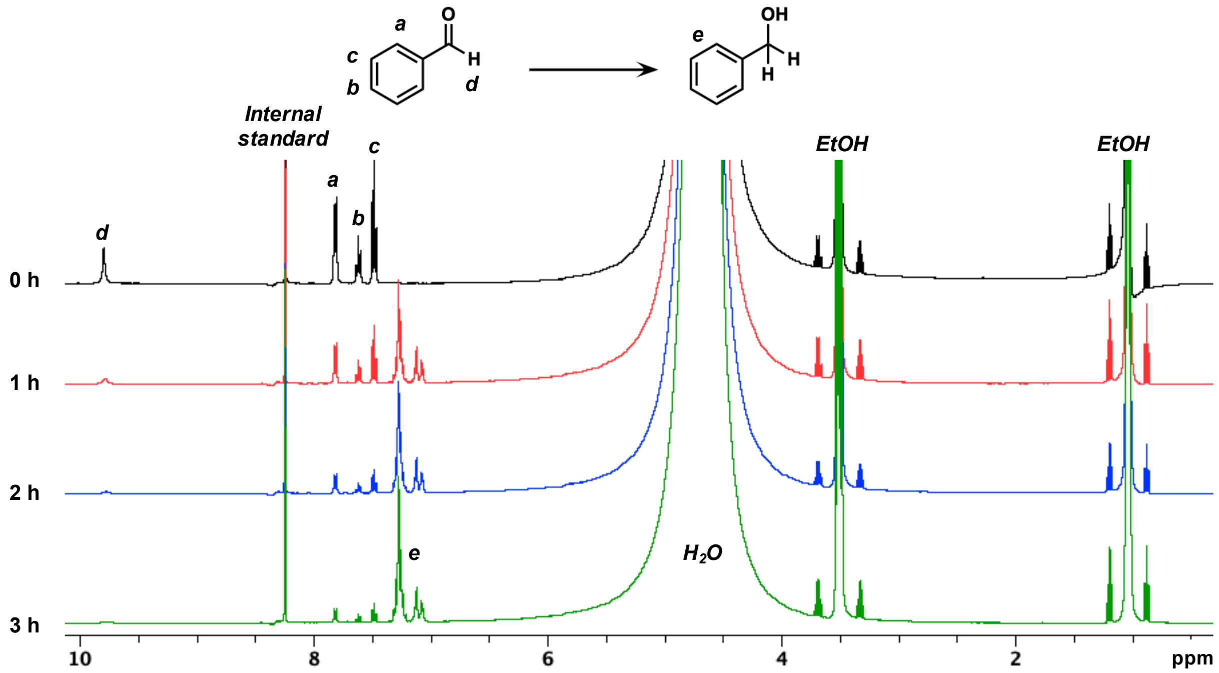

Figure S12. Crude ^{1}H-NMR spectra (64 scans) for the reaction mixtures obtained in the ECH of benzaldehyde after 0, 1, 2, and 3 hours of electrolysis. D_2O was used as the deuterated reagent for

the ^{1}H-NMR measurement. Benzene-1,3,5-tricarboxylic acid (singlet, δ = 8.3 ppm) was added as an internal standard to quantify the amount of reactant and product.

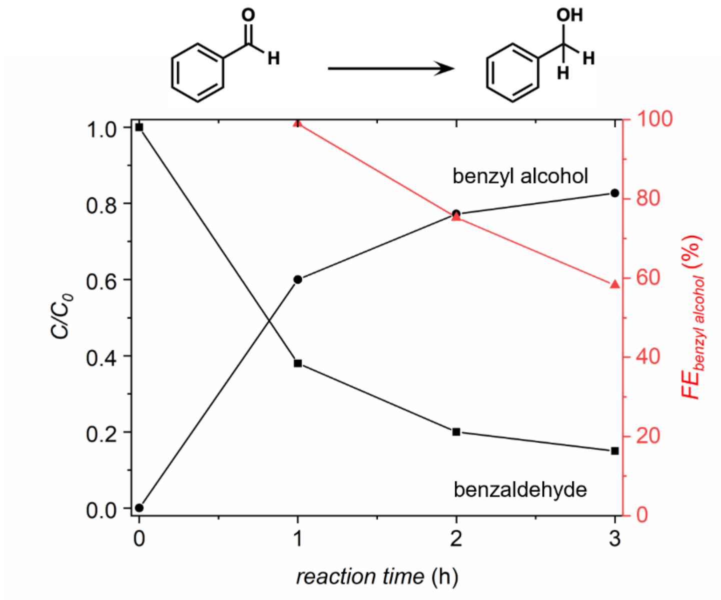

Figure S13. Conversion of benzaldehyde, yield of benzyl alcohol, and $FE_{benzyl\ alcohol}$ over 3 hours of electrolysis at –0.48 V vs RHE. Reaction conditions: Cu cathode; 10 mL of aqueous buffer solution (pH = 11.8) containing 10 vol% ethanol and 0.5 mmol of reactant.

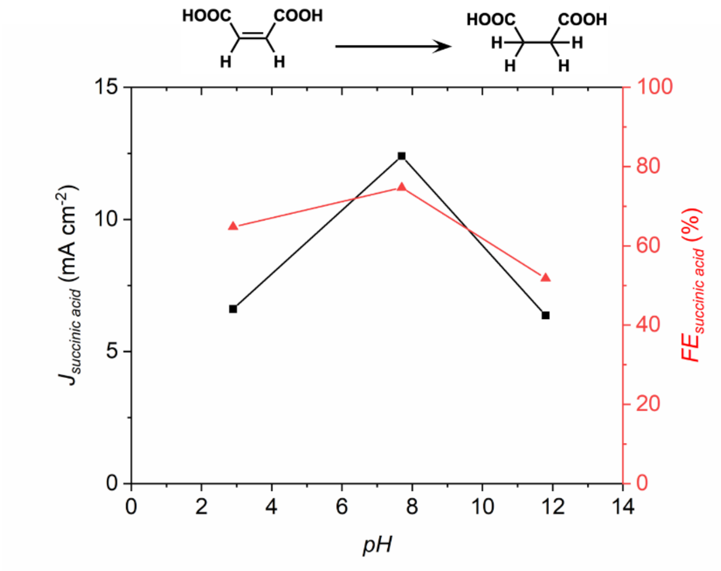

Figure S14. Optimization of the electrolyte pH for the ECH of maleic acid. $FE_{1\text{-}PEA}$ and $J_{1\text{-}PEA}$ were measured with different pH values (pH = 11.8, 7.7, and 2.9) after 1 hour of electrolysis at –0.48 V vs RHE.

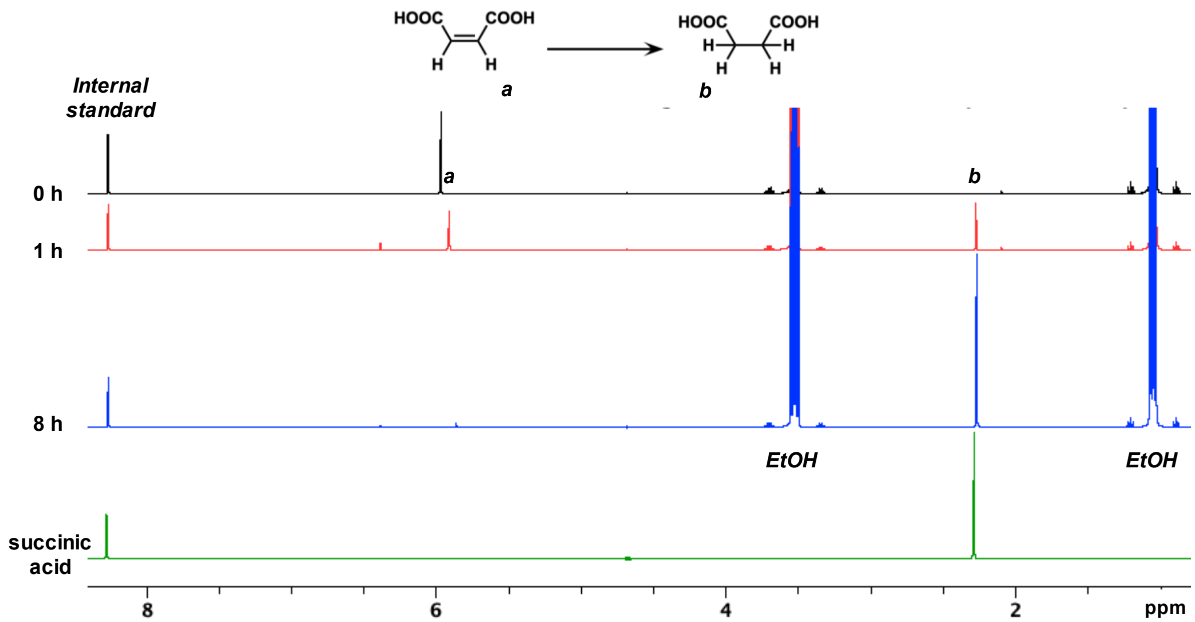

Figure S15. Crude ^{1}H-NMR spectra (64 scans, water suppression) for the reaction mixtures obtained in the ECH of maleic acid after 0, 1, and 8 hours of electrolysis. D_2O was used as the deuterated reagent for the ^{1}H-NMR measurement. Benzene-1,3,5-tricarboxylic acid (singlet, δ = 8.3 ppm) was added as an internal standard to quantify the amount of reactant and product.

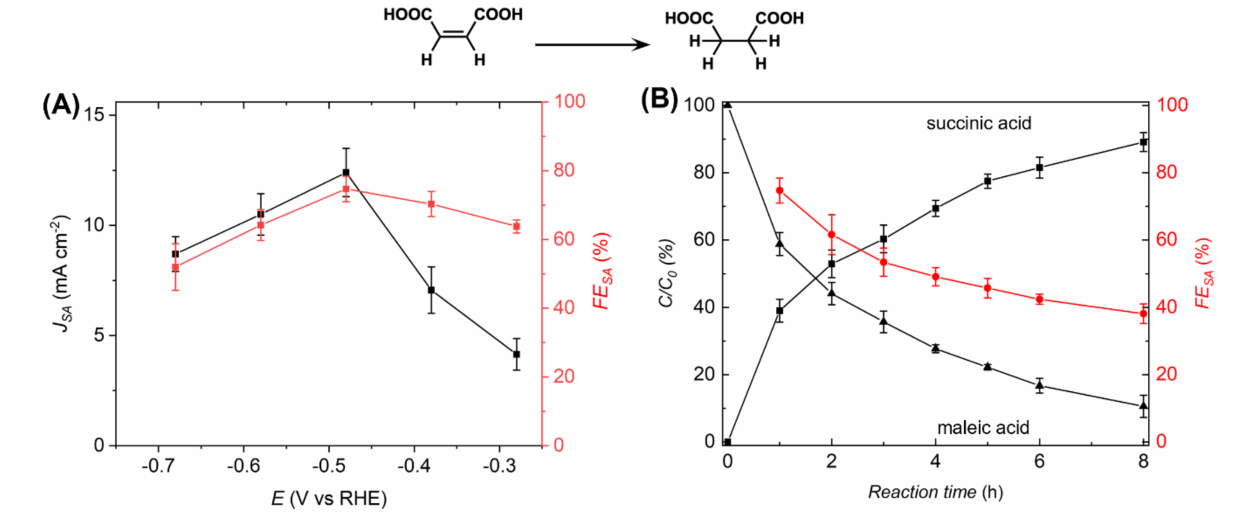

Figure S16. ECH of maleic acid to succinic acid. (**A**) FE_{SA} and J_{SA} were measured with a Cu electrocatalyst at different potentials. (**B**) Conversion of maleic acid to succinic acid on the Cu electrocatalyst over 8 hours of electrolysis at –0.48 V vs RHE. Concentrations of reactant and product were quantified by ^{1}H-NMR. Error bars correspond to the standard deviation of triplicate experiments.

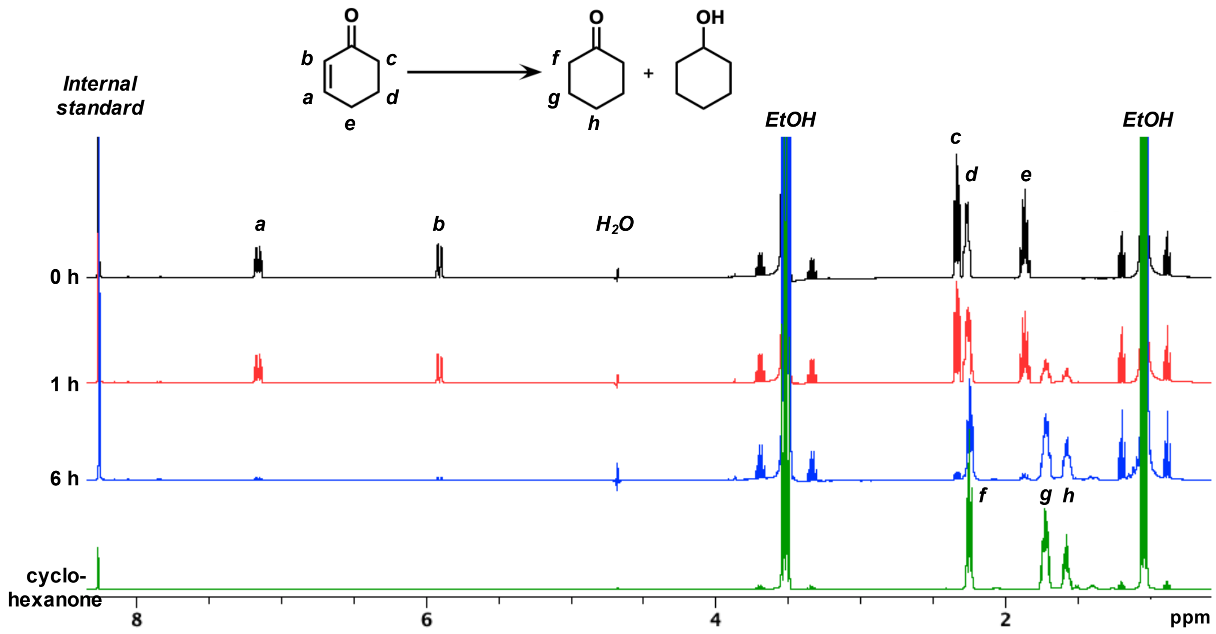

Figure S17. Crude ^{1}H-NMR spectra (64 scans, water suppression) for the reaction mixtures obtained in the ECH of cyclohex-2-en-1-one after 0, 1, and 6 hours of electrolysis. D_2O was used as the deuterated reagent for the ^{1}H-NMR measurement. Benzene-1,3,5-tricarboxylic acid (singlet, $\delta = 8.3$ ppm) was added as an internal standard to quantify the amount of reactant and products.

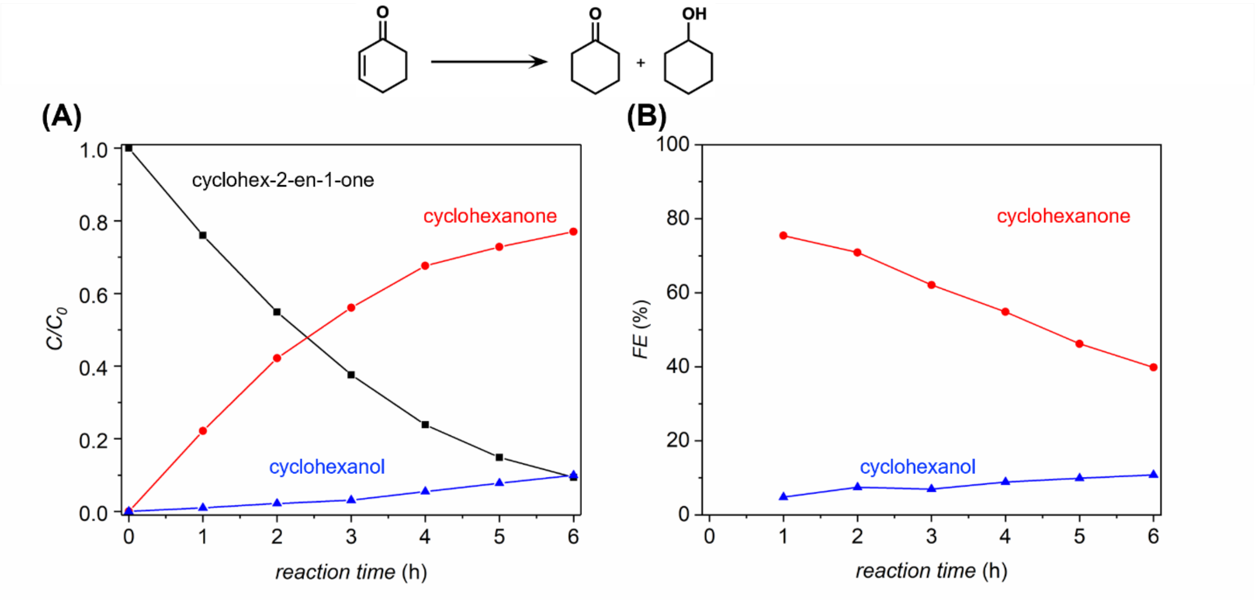

Figure S18. Conversion of cyclohex-2-en-1-one and yields (**A**) and FEs (**B**) for cyclohexanone and cyclohexanol over 6 hours of electrolysis at –0.58 V vs RHE. Reaction conditions: Cu cathode; 10 mL of aqueous buffer solution (pH = 7.7) containing 10 vol% ethanol and 0.5 mmol of reactant.

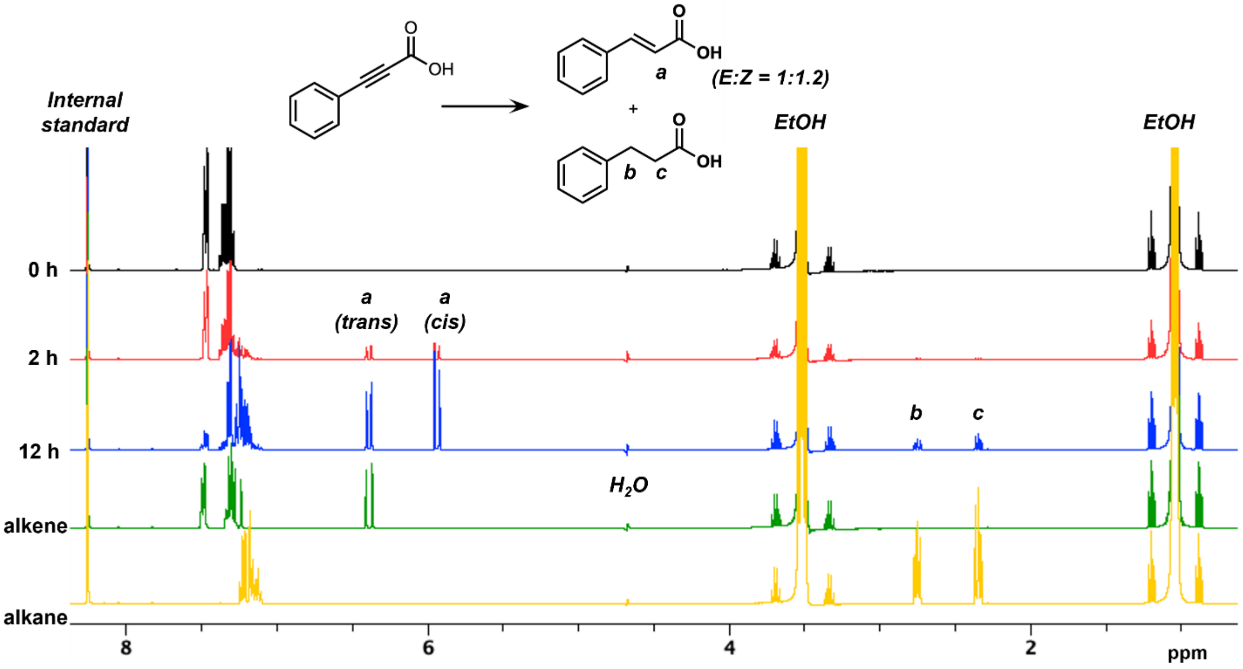

Figure S19. Crude ^{1}H-NMR spectra (64 scans, water suppression) for the reaction mixtures obtained in the ECH of phenylpropiolic acid after 0, 2, and 12 hours of electrolysis. D_2O was used as the deuterated reagent for the ^{1}H-NMR measurement. Benzene-1,3,5-tricarboxylic acid (singlet, δ = 8.3 ppm) was added as an internal standard to quantify the amount of reactant and products. The aromatic peaks of reactant and products are overlapped.

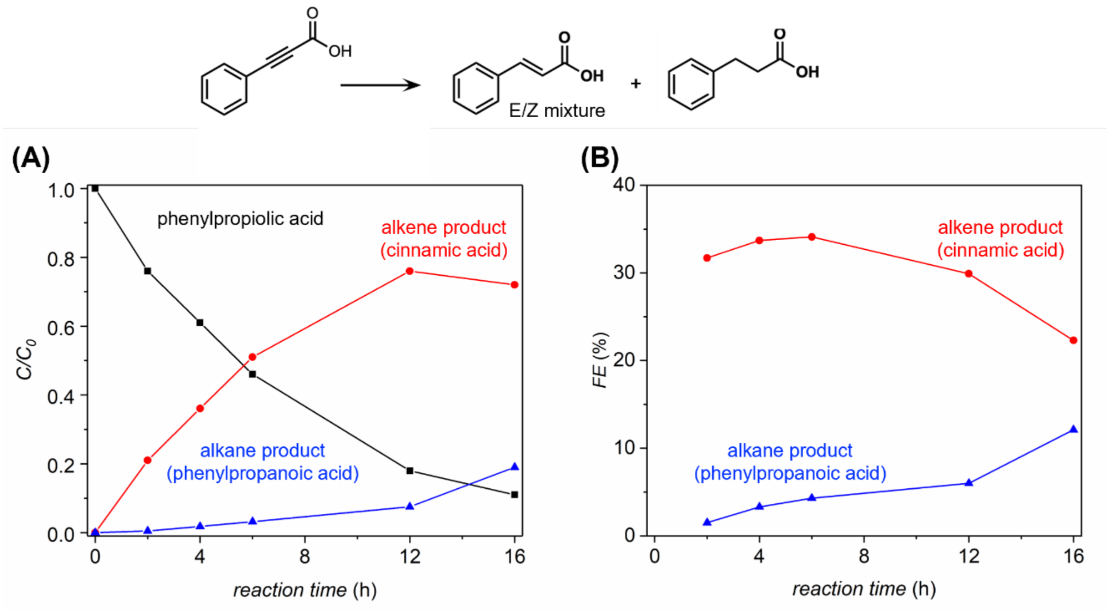

Figure S20. Conversion of phenylpropiolic acid and yields (**A**) and FEs (**B**) for the alkene (cinnamic acid) and alkane products (phenylpropanoic acid) over 16 hours of electrolysis at –0.58 V vs RHE. Reaction conditions: Cu cathode; 10 mL of aqueous buffer solution (pH = 7.7) containing 10 vol% ethanol and 0.5 mmol of reactant.

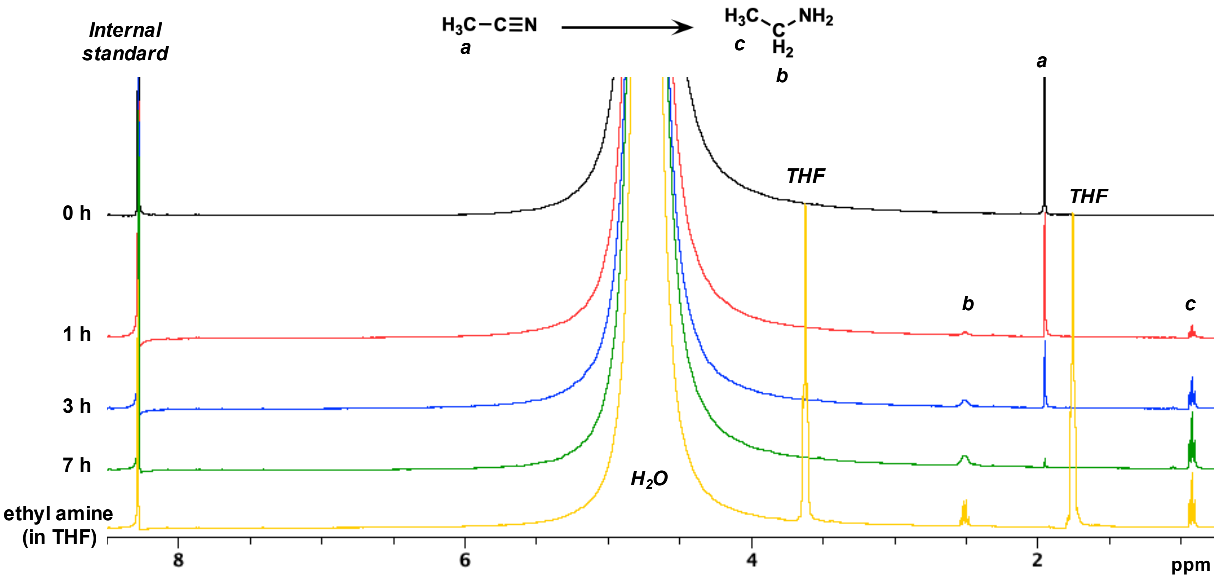

Figure S21. Crude ^{1}H-NMR spectra (64 scans) for the reaction mixtures obtained in the ECH of acetonitrile after 0, 1, 3, and 7 hours of electrolysis. D_2O was used as the deuterated reagent for the ^{1}H-NMR measurement. Benzene-1,3,5-tricarboxylic acid (singlet, $\delta = 8.3$ ppm) was added as an internal standard to quantify the amount of reactant and products. Peaks of THF were observed because the standard ethyl amine was dissolved in THF-water solution.

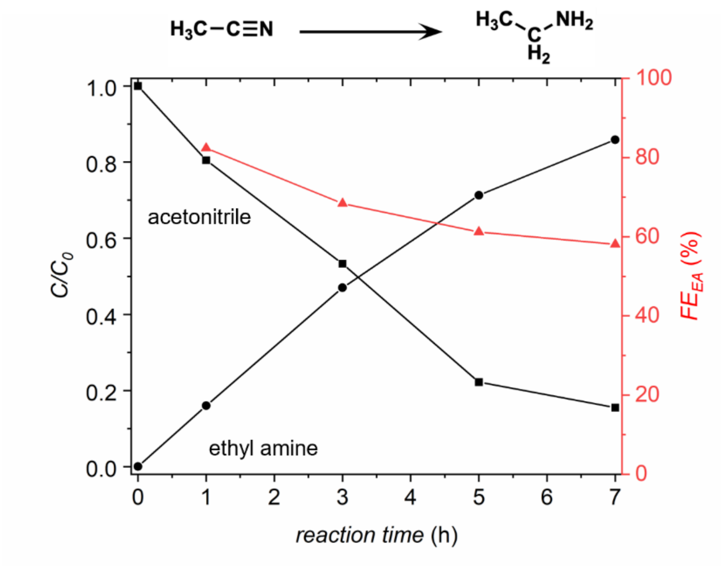

Figure S22. Conversion of acetonitrile yield of ethyl amine, and $FE_{ethyl\ amine}$ over the 7 hours of electrolysis at –0.48 V vs RHE. Reaction conditions: Cu cathode; 10 mL of aqueous buffer solution (pH = 11.8) containing 0.5 mmol of reactant.

Supplementary tables

Table S1. Calculated relative energies (ΔE) at –0.48 V_{RHE}, entropic term (TS, with T = 298 K), zero-point energy (ZPE), and heat capacity contributions (C_P for gas-phase species, C_V for adsorbates), all expressed in eV. These values were computed for the reference molecules, reaction intermediates, and the transition state involved in the ECH of AP and H_2O adsorption. ΔE values were determined using Eqs. S9-19 and S7a-S8a, where the Gibbs energy terms were substituted with electronic energies obtained from DFT simulations. The corrections upon diffusion of surface H on Cu(111) are not reported, since these were calculated for the 0.75 ML H and applied to the other systems, as described in the above section *"Modelling the ECH of AP on dynamic *H surface coverages"*.

		**ΔE**	**TS**	**ZPE**	**C_P or C_V**
Molecules	AP	-	1.11	3.69	0.24
	1-PEA	-	1.14	4.31	0.25
	H_2	-	0.40	0.30	0.09
	H_2O	-	0.58	0.58	0.10
Ag	2*H	–0.10	0.02	0.27	0.02
	*AP (O)	–0.45	0.59	3.71	0.27
	*AP (ring)	–0.61	0.69	3.70	0.28
	*AP+1*H_2O	-	0.65	4.39	0.33
	*AP+2*H_2O	-	0.91	5.04	0.43
	*AP+3*H_2O	-	1.14	5.69	0.52
	*TS (C-1H_2O)	1.53	0.73	4.45	0.34
	*TS (C-2H_2O)	0.89	0.98	5.13	0.42
	*TS (C-3H_2O)	1.21	1.26	5.77	0.51
	*TS (O)	0.10	1.01	5.10	0.44
	*APH$^•$ (C)	–0.99	0.54	4.02	0.26
	*APH$^•$ (O)	–0.42	0.56	4.01	0.27
	*1-PEA	–0.95	0.60	4.34	0.28
	*H_2O	–0.27	0.25	0.63	0.10
Au	2*H	–0.12	0.03	0.24	0.02
	*AP (O)	–0.50	0.62	3.71	0.27

	*AP (ring)	–0.71	0.56	3.71	0.26
	*AP+2*H_2O	-	0.90	5.05	0.42
	*TS	–0.20	0.99	5.12	0.43
	*APH• (C)	–0.53	0.52	4.03	0.25
	*APH• (O)	–0.81	0.58	4.02	0.27
	*1-PEA	–2.00	0.58	4.34	0.28
	*H_2O	–0.29	0.26	0.63	0.11
Cu (uniform 0.75 ML H)	*AP (O)	–0.52	0.58	3.72	0.26
	*AP (ring)	–0.55	0.55	3.72	0.26
	*AP+2*H_2O	-	0.84	5.05	0.43
	*TS	0.05	0.89	5.16	0.41
	*APH• (C)	–0.59	0.51	4.04	0.25
	*APH• (O)	–0.43	0.61	4.00	0.28
	*1-PEA	–1.65	0.60	4.34	0.28
	2*H	–1.95	0.66	4.34	0.28
	*H_2O	–0.16	0.32	0.61	0.12
Ni	*AP (O)	–0.31	0.58	3.72	0.26
	*AP (ring)	–0.61	0.56	3.72	0.26
	*APH• (C)	0.80	0.51	4.02	0.25
	*APH• (O)	–0.12	0.61	4.02	0.27
	*1-PEA	–0.75	0.54	4.35	0.27
	2*H	–1.95	0.66	4.33	0.28
	*H_2O	–0.13	0.32	0.61	0.12
Pt	*AP (O)	-	-	-	-
	*AP (ring)	–0.60	0.54	3.72	0.26
	*APH• (C)	0.48	0.52	4.02	0.25
	*APH• (O)	–0.42	0.59	4.03	0.27
	*1-PEA	–1.07	0.64	4.33	0.28
	2*H	–1.96	0.65	4.33	0.28
	*H_2O	–0.15	0.400	0.61	0.13

Table S2. Electronic energies, enthalpy corrections, and resulting enthalpy values for H_2, O_2, H_2O, AP, 1-PEA, and a graphene unit cell consisting of two carbon atoms, computed at 298 K (in eV). The enthalpy (*H*) was determined as:

$$H = E_{el} + H_{corr}$$

	**H_2**	**O_2**	**H_2O**	**AP**	**1-PEA**	**Graphene**
E_{el}	–7.470	–9.843	–14.721	–113.489	–121.362	–19.202
H_{corr}	+0.389	+0.187	+0.680	+3.926	+4.567	-
H	–7.081	–9.656	–14.041	–109.563	–116.795	–19.202

Supplementary references

(1) Kresse, G.; Furthmüller, J. Efficient Iterative Schemes for *Ab Initio* Total-Energy Calculations Using a Plane-Wave Basis Set. *Phys. Rev. B,* **1996**, *54* (16), 11169–11186.

(2) Wellendorff, J.; Lundgaard, K. T.; Møgelhøj, A.; Petzold, V.; Landis, D. D.; Nørskov, J. K.; Bligaard, T.; Jacobsen, K. W. Density Functionals for Surface Science: Exchange-Correlation Model Development with Bayesian Error Estimation. *Phys. Rev. B,* **2012**, *85* (23), 235149.

(3) Blöchl, P. E. Projector Augmented-Wave Method. *Phys. Rev. B,* **1994**, *50* (24), 17953–17979.

(4) Methfessel, M.; Paxton, A. T. High-Precision Sampling for Brillouin-Zone Integration in Metals. *Phys. Rev. B,* **1989**, *40* (6), 3616–3621.

(5) Hjorth Larsen, A.; Jørgen Mortensen, J.; Blomqvist, J.; Castelli, I. E.; Christensen, R.; Dułak, M.; Friis, J.; Groves, M. N.; Hammer, B.; Hargus, C.; Hermes, E. D.; Jennings, P. C.; Bjerre Jensen, P.; Kermode, J.; Kitchin, J. R.; Leonhard Kolsbjerg, E.; Kubal, J.; Kaasbjerg, K.; Lysgaard, S.; Bergmann Maronsson, J.; Maxson, T.; Olsen, T.; Pastewka, L.; Peterson, A.; Rostgaard, C.; Schiøtz, J.; Schütt, O.; Strange, M.; Thygesen, K. S.; Vegge, T.; Vilhelmsen, L.; Walter, M.; Zeng, Z.; Jacobsen, K. W. The Atomic Simulation Environment—a Python Library for Working with Atoms. *J. Phys. Condens. Matter.* **2017**, *29* (27), 273002.

(6) Jain, A.; Ong, S. P.; Hautier, G.; Chen, W.; Richards, W. D.; Dacek, S.; Cholia, S.; Gunter, D.; Skinner, D.; Ceder, G.; Persson, K. A. Commentary: The Materials Project: A Materials Genome Approach to Accelerating Materials Innovation. *APL Mater.* **2013**, *1* (1), 011002.

(7) Birch, F. Finite Elastic Strain of Cubic Crystals. *Phys. Rev.* **1947**, *71* (11), 809–824.

(8) Barmparis, G. D.; Lodziana, Z.; Lopez, N.; Remediakis, I. N. Nanoparticle Shapes by Using Wulff Constructions and First-Principles Calculations. *Beilstein J. Nanotechnol.* **2015**, *6*, 361–368.

(9) Rahm, J.; Erhart, P. WulffPack: A Python Package for Wulff Constructions. *J. Open Source Softw.* **2020**, *5* (45), 1944.

(10) Brogaard, R. Y.; Henry, R.; Schuurman, Y.; Medford, A. J.; Moses, P. G.; Beato, P.; Svelle, S.; Nørskov, J. K.; Olsbye, U. Methanol-to-Hydrocarbons Conversion: The Alkene Methylation Pathway. *J. Catal.* **2014**, *314*, 159–169.

(11) Heyden, A.; Bell, A. T.; Keil, F. J. Efficient Methods for Finding Transition States in Chemical Reactions: Comparison of Improved Dimer Method and Partitioned Rational Function Optimization Method. *J. Chem. Phys.* **2005**, *123* (22), 224101.

(12) H. Jónsson, G. Mills, K. W. Jacobsen. Nudged Elastic Band Method for Finding Minimum Energy Paths of Transitions. In *Classical and Quantum Dynamics in Condensed Phase Simulations*; World Scientific Publishing, 1998; pp 385–404.

(13) Nørskov, J. K.; Rossmeisl, J.; Logadottir, A.; Lindqvist, L.; Kitchin, J. R.; Bligaard, T.; Jónsson, H. Origin of the Overpotential for Oxygen Reduction at a Fuel-Cell Cathode. *J. Phys. Chem. B,* **2004**, *108* (46), 17886–17892.

(14) Urrego-Ortiz, R.; Builes, S.; Illas, F.; Calle-Vallejo, F. Gas-Phase Errors in Computational Electrocatalysis: A Review. *EES Catal.* **2024**, *2* (1), 157–179.

(15) *The National Institute for Standards and Technology Chemistry WebBook (Acetophenone)*. https://webbook.nist.gov/cgi/cbook.cgi?ID=C98862&Mask=1#Thermo-Gas (accessed 2025-01-06).

(16) *Chemical Properties of Benzenemethanol, «alpha»-methyl- (CAS 98-85-1)*. Cheméo. https://www.chemeo.com/cid/16-106-6/Benzenemethanol-alpha-methyl (accessed 2025-01-06).

(17) *The National Institute for Standards and Technology Chemistry WebBook (water)*. https://webbook.nist.gov/cgi/cbook.cgi?ID=C7732185&Mask=1#Thermo-Gas (accessed 2025-01-06).

(18) Urrego-Ortiz, R.; Builes, S.; Calle-Vallejo, F. Fast Correction of Errors in the DFT-Calculated Energies of Gaseous Nitrogen-Containing Species. *ChemCatChem* **2021**, *13* (10), 2508–2516.

(19) Bondue, C. J.; Koper, M. T. M. Electrochemical Reduction of the Carbonyl Functional Group: The Importance of Adsorption Geometry, Molecular Structure, and Electrode Surface Structure. *J. Am. Chem. Soc.* **2019**, *141* (30), 12071–12078.

(20) Motiar Rahaman, Virgil Andrei, Demelza Wright, Erwin Lam, Chanon Pornrungroj, Subhajit Bhattacharjee, Christian M. Pichler, Heather F. Greer, Jeremy J. Baumberg, Erwin Reisner. Solar-Driven Liquid Multi-Carbon Fuel Production Using a Standalone Perovskite–BiVO4 Artificial Leaf. *Nat. Energy*, **2023**, *8*, 629–638.
